# CO_2_ fertilization of terrestrial photosynthesis inferred from site to global scales

**DOI:** 10.1073/pnas.2115627119

**Published:** 2022-03-01

**Authors:** Chi Chen, William J. Riley, I. Colin Prentice, Trevor F. Keenan

**Affiliations:** ^a^Climate and Ecosystem Sciences Division, Lawrence Berkeley National Laboratory, Berkeley, CA 94720;; ^b^Department of Environmental Science, Policy and Management, University of California, Berkeley, CA 94720;; ^c^Department of Life Sciences, Imperial College London, Ascot SL5 7PY, United Kingdom

**Keywords:** CO_2_ fertilization effect, photosynthesis, GPP, optimization theory, carbon and water coupling

## Abstract

Global photosynthesis is increasing with elevated atmospheric CO_2_ concentrations, a response known as the CO_2_ fertilization effect (CFE), but the key processes of CFE are not constrained and therefore remain uncertain. Here, we quantify CFE by combining observations from a globally distributed network of eddy covariance measurements with an analytical framework based on three well-established photosynthetic optimization theories. We report a strong enhancement of photosynthesis across the observational network (9.1 gC m^−2^ year^−2^) and show that the CFE is responsible for 44% of the gross primary production (GPP) enhancement since the 2000s, with additional contributions primarily from warming (28%). Soil moisture and specific humidity are the two largest contributors to GPP interannual variation through their influences on plant hydraulics. Applying our framework to satellite observations and meteorological reanalysis data, we diagnose a global CO_2_-induced GPP trend of 4.4 gC m^−2^ year^−2^, which is at least one-third stronger than the median trends of 13 dynamic global vegetation models and eight satellite-derived GPP products, mainly because of their differences in the magnitude of CFE in evergreen broadleaf forests. These results highlight the critical role that CFE has played in the global carbon cycle in recent decades.

## Significance

The magnitude of the CO_2_ fertilization effect on terrestrial photosynthesis is uncertain because it is not directly observed and is subject to confounding effects of climatic variability. We apply three well-established eco-evolutionary optimality theories of gas exchange and photosynthesis, constraining the main processes of CO_2_ fertilization using measurable variables. Using this framework, we provide robust observationally inferred evidence that a strong CO_2_ fertilization effect is detectable in globally distributed eddy covariance networks. Applying our method to upscale photosynthesis globally, we find that the magnitude of the CO_2_ fertilization effect is comparable to its in situ counterpart but highlight the potential for substantial underestimation of this effect in tropical forests for many reflectance-based satellite photosynthesis products.

Vegetation photosynthesis is responsible for the largest flux of carbon from the atmosphere into the biosphere (1). Both theory and experimental observations show that the resulting gross primary production (GPP) increases with rising atmospheric CO_2_, a process known as the CO_2_ fertilization effect (CFE) (2–8). The CFE plays a strong role in offsetting anthropogenic emissions by directly increasing the terrestrial carbon assimilation rate. It affects both the global carbon (6) and water budgets (9), which leads to changes in temperature and precipitation, and indirectly affects climate change through vegetation–climate feedbacks (10) by increasing plant growth (11). Understanding the CFE is thus critical to understanding the evolution of Earth’s climate.

Despite the importance of global photosynthesis, however, there are few long-term records of observationally inferred GPP in natural environments. This leads to large uncertainty in the magnitude of photosynthetic change over time and the CFE (4, 12–14). Eddy covariance (EC), a measurement of gas exchange deployed at hundreds of ecosystems worldwide, provides estimates of photosynthesis that could contain information on the CFE (14). Estimating CFE from such observations is challenging, however, because of the large confounding effects from climatic variability of natural environments and the short duration of measurements at many sites. Studies that have attempted to isolate the CFE from carbon fluxes have been limited either to simple statistical approaches that preclude causative attribution (15, 16) or to process-based approaches that, despite capturing the sign of responses, report widely varying sensitivities (17, 18). This uncertainty has led to debate regarding the magnitude of the CFE, as evidenced by the large spread between satellite- and Earth system model (ESM)–based CFE estimates (19–23). Indeed, because of the lack of observational evidence at the ecosystem scale, many GPP products based on satellite-derived metrics do not explicitly account for CFE (20, 24, 25), while current representations of CFE also vary markedly among ESMs (26–28).

Here, we leverage globally distributed EC observations and reanalysis data to detect and attribute CFE using an eco-evolutionary optimality (EEO) framework. This framework reconciles three well-established optimization theories in combination with Fick’s law and the Farquhar-von Caemmerer-Berry (FvCB) photosynthesis model (29), constraining photosynthesis and key intermediate variables, namely stomatal conductance (*g*) (30, 31), intercellular leaf CO_2_ concentration (*c_i_*) (32, 33), and photosynthetic capacity (34–38) (see *Materials and Methods* for general descriptions and SI Appendix for derivations). The framework can analytically diagnose GPP and its sensitivity to seven measurable variables, namely atmospheric CO_2_ concentration (*c_a_*), leaf area index (LAI), air temperature (*T_a_*), volumetric soil water content (SWC), specific humidity (*q_a_*), incident shortwave radiation (*SW_in_*), and surface pressure (*P*), without the need to prescribe biome-specific photosynthetic properties (35). Our results show an overall increase in photosynthesis at measurement sites worldwide and attribute a large proportion of the increase to the effect of rising CO_2_.

## Strong CO_2_-Induced Increase in Site-Scale Photosynthesis.

Three metrics are used to quantify and assess the response of GPP to CO_2_.

• β_CO2_ (gC m^−2^ year^−1^ ppmv^−1^) is the partial differential sensitivity of GPP to CO_2_ for given environmental conditions (i.e., $\frac{\partial \mathrm{GPP}}{\partial c_{a}}$ in Eq. **1**). It is directly diagnosed by the EEO framework, which decouples the confounding effects on GPP from other environmental conditions.
• ${\text{β}}_{\mathrm{app}}^{\text{ln}}$ is a dimensionless, apparent logarithmic GPP response ratio to CO_2_ following Ref. 8 for comparison with other studies (Eq. **3**). Changes in GPP include both CO_2_ and non-CO_2_ effects.
• ${\text{β}}_{\mathrm{dir}}^{\text{ln}}$ is a dimensionless, direct logarithmic GPP response ratio to CO_2_, which means changes in GPP include CO_2_ effects only. Confounding effects are neglected or analytically decoupled through ideal experimental manipulations such as free-air CO_2_ enrichment (FACE) experiments or partial derivative approaches. It shares the same equation as ${\text{β}}_{\mathrm{app}}^{\text{ln}}$, and is only used for comparison with other studies.

**Fig. 1.**
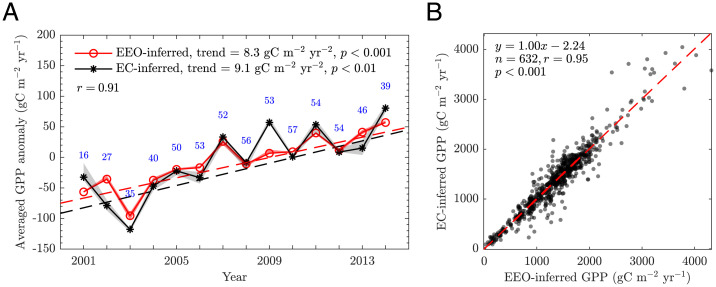
The EEO-inferred GPP reproduces the trend and IAV of site-scale EC-inferred GPP. (*A*) Trends and interannual variations. GPP anomaly is defined as the deviation from climatological mean during the study period, further averaged across all 68 sites (see anomalies for individual sites in *SI Appendix*, Fig. S1). Blue numbers indicate the number of sites with available GPP. The shaded area represents the range of variation due to partitioning and friction velocity filtering methods in EC-inferred GPP (gray), and their corresponding variability propagated to the EEO-inferred GPP through canopy upscaling calibration (light red). Solid lines are the ensemble mean of EC-inferred GPP based on the above methods (black) and the resulting EEO-inferred GPP (red). (*B*) Scatter plot of EEO-inferred and EC-inferred GPP. Dashed lines indicate best-fit lines.

**Fig. 2.**
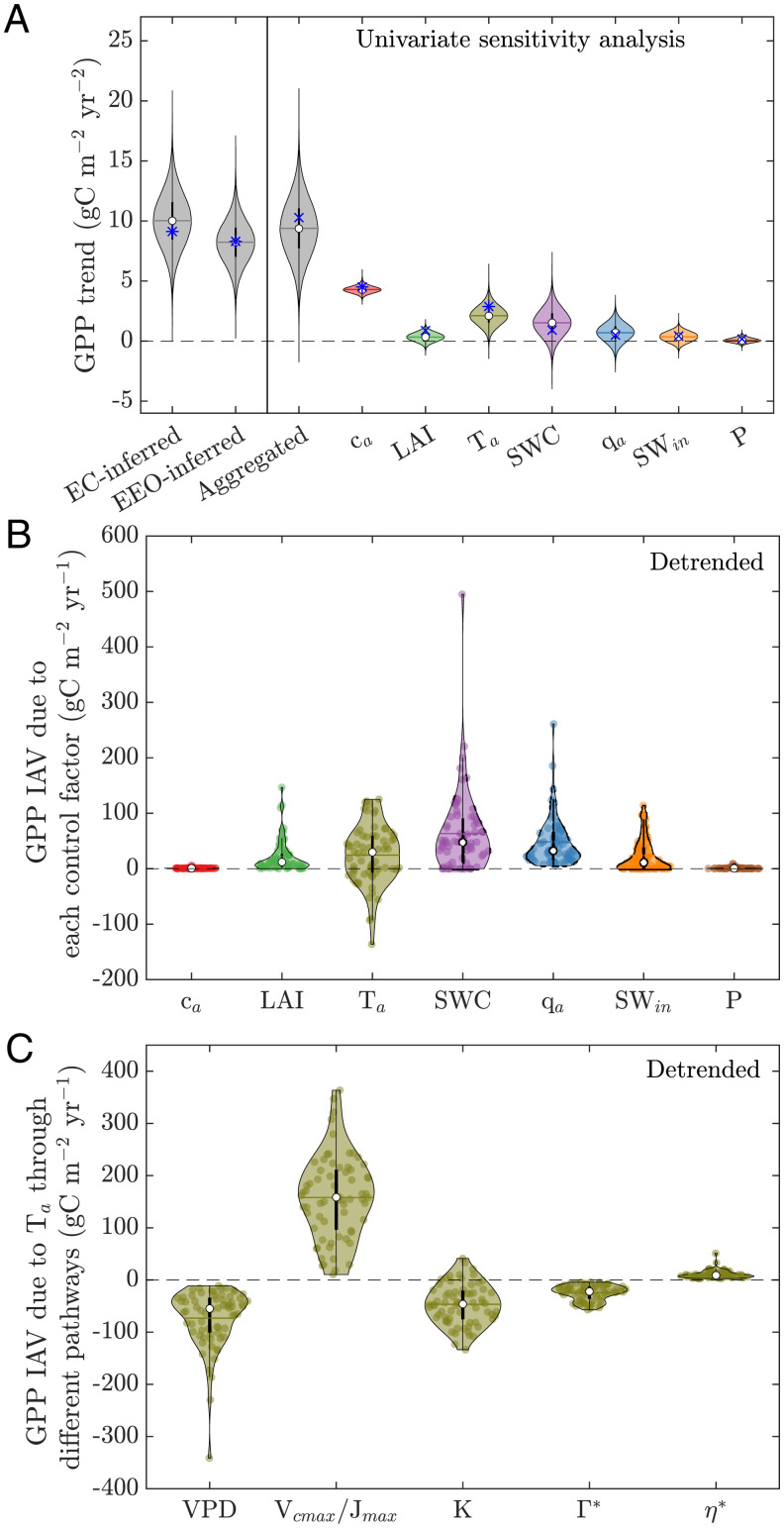
GPP trend and IAV are dominated by different factors at site-scale. (*A*) Probability density of GPP trends by 50% resampling of site-years for each site for 200,000 times (see illustration in *SI Appendix*, Fig. S3*D*). Attribution of GPP trends is computed by univariate sensitivity analysis. The other variables are kept as the climatological mean of their study period. Blue * and × indicate statistically significant (*P* < 0.05) and insignificant trends in the estimated GPP, respectively. The hollow circles, thick black vertical lines, and gray horizontal lines are the median, IQR, and mean of trends. “EC-inferred”: GPP trends estimated from the EC network. “EEO-inferred”: GPP trends directly estimated by the EEO framework. “Aggregated”: sum of GPP trends for each factor from the sensitivity analysis. (*B*) GPP IAV is proxied by the product of partial derivatives of GPP to control factors and IAVs of control factors. Partial derivatives are diagnosed using climatological means of the control factors and IAVs are defined as 1 SD of the data with long-term trends removed. (*C*) GPP IAV induced by temperature IAV through different pathways. *K* is the Michaelis-Menten coefficient, Γ* is the CO_2_ compensation point, and η* is the ratio of water viscosity under ambient air temperature to a reference temperature at 298.15 K. In *B* and *C*, each violin consists of values from 68 sites.

We find a strong increasing trend of GPP measured across 632 site-years of observations in the EC network of 9.1 gC m^−2^ year^−2^ between 2001 and 2014 [interquartile range (IQR) ∈ [8.5, 11.5], *P* < 0.01, ${\text{β}}_{\mathrm{app}}^{\text{ln}}$ = 1.24]. This EC-inferred trend is captured by our EEO framework (8.3 gC m^−2^ year^−2^, IQR ∈ [7.0, 9.4], *P* < 0.01, ${\text{β}}_{\mathrm{app}}^{\text{ln}}$ = 1.12) (Figs. 1*A* and 2*A*). Both ${\text{β}}_{\mathrm{app}}^{\text{ln}}$ values are relatively high compared with expectations from measured direct responses to CO_2_ (i.e., ${\text{β}}_{\mathrm{dir}}^{\text{ln}}$) but are similar to several published indirect apparent GPP proxies (8): carbonyl sulfide records (${\text{β}}_{\mathrm{app}}^{\text{ln}}$ = 0.95) (39), ice-core measurements of atmospheric O_2_ isotopes (${\text{β}}_{\mathrm{app}}^{\text{ln}}$ = 1.3) (40), and satellite monitoring of water-use efficiency (${\text{β}}_{\mathrm{app}}^{\text{ln}}$ = 1.1) (41). Since ${\text{β}}_{\mathrm{app}}^{\text{ln}}$ is derived from natural environments and influenced by CO_2_ and climate variability, it differs from the direct ${\text{β}}_{\mathrm{dir}}^{\text{ln}}$ discussed later. The detected overall trends are robust to the uncertainty caused by filtering of low-quality data, and the uneven distribution of sites and site-years (*SI Appendix*, Fig. S2). Variations in the EC-inferred GPP due to partitioning methods and friction velocity filtering methods have a minimal effect on the EEO-inferred GPP (shaded area in Fig. 1*A*), where the former is used to calibrate the canopy upscaling of the latter. In general, the EEO-inferred GPP reproduces the interannual variability (IAV) of the EC-inferred GPP (Pearson’s *r* = 0.91) (Fig. 1*A*; *SI Appendix*, Fig. S1). The high level of agreement from annual (Fig. 1*B*) to monthly (*SI Appendix*, Fig. S3) scales supports the use of the EEO framework for attribution analysis.

To quantify the GPP trends contributed by different factors, we use the EEO framework to perform a univariate sensitivity analysis (*SI Appendix*, Fig. S4). The aggregated trend from individual factors (10.3 gC m^−2^ year^−2^, IQR ∈ [7.7, 11.0]) is similar to the EEO-inferred and EC-inferred trends (Fig. 2*A*). We find a strong direct contribution from *c_a_* at the site level, which accounts for 44% (4.5 gC m^−2^ year^−2^, *P* < 0.001) of the aggregated GPP trend (Fig. 2*A*). We also diagnosed another overall CO_2_-induced GPP trend using the partial differential approach (4.9 gC m^−2^ year^−2^, the first term on the right-hand side of Eq. **1**), resulting in a small difference (0.4 gC m^−2^ year^−2^) compared with the univariate analysis. We then convert the estimate from the univariate analysis, which corresponds to a direct response ratio (${\text{β}}_{\mathrm{dir}}^{\text{ln}}$) of 0.61 for GPP to CO_2_. This ${\text{β}}_{\mathrm{dir}}^{\text{ln}}$ is lower than those light-saturated leaf-level analyses in FACE experiments (${\text{β}}_{\mathrm{dir}}^{\text{ln}}$ = 0.79) (4, 8) and those using deuterium isotopomers (${\text{β}}_{\mathrm{dir}}^{\text{ln}}$ = 1.0) (8, 42) but is comparable to a theoretical ${\text{β}}_{\mathrm{dir}}^{\text{ln}}$ of 0.6 in Ref. 8 that considers canopy radiative transfer, suggesting a coexisting of light-saturated and light-limited photosynthesis over our study sites. CFE is stronger under light-saturated conditions but is present regardless of light conditions (27, 28). An increase in *c_a_* eventually elevates leaf *c_i_* through optimization (*SI Appendix*, Eq. **S16**), which in the FvCB scheme leads to higher light use efficiency and carbon assimilation rates (7, 32).

In addition to *c_a_*, *T_a_* plays the second most important role, accounting for 28% (2.9 gC m^−2^ year^−2^, *P* < 0.05) of the aggregated trend (Fig. 2*A*), mainly because of the effect of warming on photosynthetic capacity, i.e., the maximum rate of Rubisco activity, *V_cmax_*, and the maximum rate of electron transport, *J_max_* (Fig. 2*C*) (38). Although increasing temperature negatively affects GPP through, for example, vapor pressure deficit (VPD) and some biochemical reaction pathways (Fig. 2*C*), these negative effects are weaker than the positive effects of temperature (Fig. 2 *A* and *B*). SWC and specific humidity, as proxies for plant water supply and atmospheric water demand, together contribute 14% of the aggregated trend (0.91 and 0.50 gC m^−2^ year^−2^, *P* > 0.05) but are statistically insignificant. These two factors (i.e., SWC and *q_a_*), however, are the largest contributors to GPP IAV, and their contributions (median values are 48 and 33 gC m^−2^ year^−1^, respectively; Fig. 2*B*) are an order of magnitude larger than those of the GPP trends (Fig. 2*A*). These results are consistent with previous data and ESM-driven studies on plant responses to water (43–47) and are further supported by observations that GPP anomalies are regulated by stomatal conductance and leaf *c_i_*, as plants attempt to maximize their efficiency of carbon gain per unit water loss (30, 32, 48, 49).

Other factors, such as long-term changes in LAI and radiation, could also lead to changes in GPP across the EC network. However, we find a small and statistically insignificant trend in GPP due to increased LAI (0.88 gC m^−2^ year^−2^; Fig. 2*A*), which is consistent with a previous study that solely considered the response of GPP to changes in LAI (50). This small LAI-induced trend in GPP is comparable to the LAI trend, both of which are on the order of ‰ year^−1^ (51), although our framework diagnoses a large underlying sensitivity of GPP to LAI (mean $\frac{\partial \mathrm{GPP}}{\partial \mathrm{LAI}}$ = 119, intersite SD = 178, unit: gC m^−2^ year^−1^). In addition, light saturation in canopy radiative transfer (52) may lead to a low LAI-induced GPP trend as leaf area increases do not effectively translate to increases in the fraction of absorbed photosynthetically active radiation at high LAI (3, 20). For the changes in incoming shortwave radiation, their contribution to the aggregated GPP is also small (0.43 gC m^−2^ year^−2^; Fig. 2*A*) since there is no large trend in radiation over the study sites at the annual scale. Therefore, we conclude that neither changes in LAI nor radiation significantly contribute to the observed upward trend in GPP. The effect of changes in surface pressure is also negligible (Fig. 2 *A* and *B*).

## Effects of CO_2_ Fertilization at the Global Scale.

**Fig. 3.**
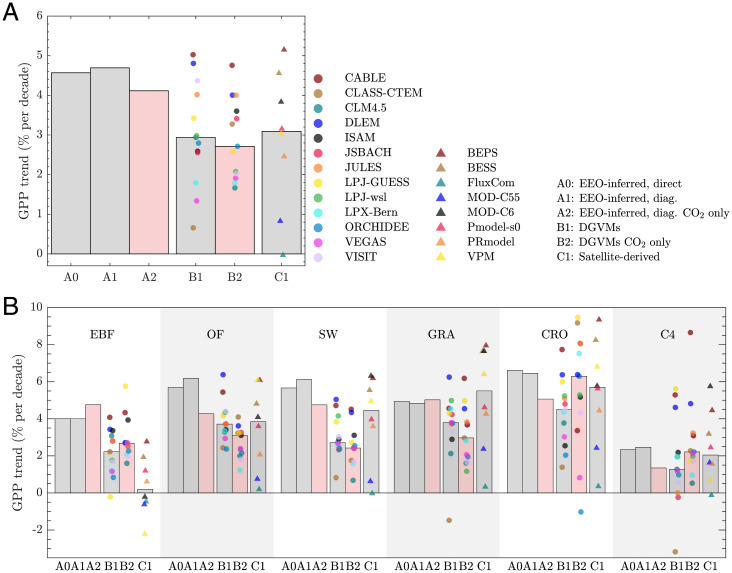
Trends in EEO-inferred, DGVM, and satellite-derived GPP at the global and ecosystem scales relative to their respective mean GPP from 2001 to 2016. (*A*) All biomes. (*B*) Individual biomes, including EBFs, other forests (OF), short woody vegetation (SW), grasslands (GRA), croplands (CRO), and C4 vegetation (C4). “A0”: estimated directly from our EEO framework. “A1”: diagnostic trends composited from partial derivatives using the EEO framework. “A2”: diagnostic CO_2_-induced trends through the partial derivative approach using the EEO framework. “B1”: 13 DGVM models, all forcing time-varying. “B2”: 13 DGVM models, CO_2_ only. “C1”: eight satellite-derived GPP products. For B1, B2, and C1, the bars represent their median. The gray bars represent GPP trends considering all effects. The red bars represent GPP trends caused by CO_2_ only. BEPS, Boreal Ecosystem Productivity Simulator; BESS, Breathing Earth System Simulator.

To examine global CFE patterns, we apply our framework to Moderate Resolution Imaging Spectroradiometer (MODIS) LAI and European Centre for Medium-Range Weather Forecasts Reanalysis Version 5 – Land data (hereafter, ERA5-land). Here, our EEO framework is calibrated by the ensemble mean of all eight satellite-derived GPP products. We then assess both the GPP trend directly estimated by the EEO framework and the underlying differential sensitivities, which themselves can be used to reconstruct a composite trend estimate (Eq. **1**). The two approaches show almost identical apparent GPP trends of about 4.7% decade^−1^ (*P* < 0.001) relative to their mean GPP throughout 2001 and 2016 (A0 and A1 in Fig. 3*A*). The resulting CO_2_-induced GPP trend is about 4.1% decade^−1^ (4.4 gC m^−2^ year^−2^, *P* < 0.001) (A2 in Fig. 3*A*; *SI Appendix*, Fig. S5). We note that the fractional contribution of *c_a_* to the apparent GPP trend at the global-scale analysis may be uncertain, and we do not report it because of the uncertainties in apparent GPP trend caused by the uncertain trends in climate forcings. However, estimates of the CO_2_-induced GPP trends (A2 in Fig. 3, calculated as the product of β_CO2_ and Δ*c_a_*) are independent of the uncertainties in the climate forcing trends because of the nature of our partial differential approach: 1) The spatial variation of β_CO2_ is rational because the climate forcings well capture their spatial variations at the global scale (53–55), and 2) Δ*c_a_* is derived from the National Oceanic and Atmospheric Administration (NOAA)’s observations with high confidence. As a result, after excluding the spatial heterogeneity introduced by LAI and canopy structure, we show a good agreement when comparing the leaf-level CO_2_-induced GPP trend at the global-scale analysis with their EC site counterparts (*r* = 0.88; *SI Appendix*, Fig. S6*A*).

We compare our CO_2_-induced GPP trends (A2 in Fig. 3) with simulations from 13 dynamic global vegetation models (DGVMs) and eight satellite-derived GPP products during their overlap period (2001 to 2016). Our estimates are at the upper end of their trend distribution, at least one-third stronger than their median (Fig. 3*A*). The lower GPP trends in the median of DGVMs (B1) and satellite-derived products (C1) is likely due to their smaller CFE in evergreen broadleaf forests (EBFs) (Fig. 3*B*). Our EEO framework suggests similar relative GPP trends caused by CO_2_ among all C3 species (including EBFs), ranging from 4.3 to 5.1% decade^−1^ (A2 in Fig. 3*B*). We further replace the ensemble mean of all satellite-derived GPP with each individual product to calibrate the EEO framework, finding a small spread in the diagnosed CO_2_-induced relative GPP trends (*SI Appendix*, Table S2), despite the fact that these individual satellite GPP products used for calibration vary markedly (global GPP in 2001 with a minimum of MODIS Collection 5.5 [MOD-C55] at 104 Pg C year^−1^ and a maximum of Pmodel-s0 at 131 Pg C year^−1^). In EBFs, it is noteworthy that the satellite GPP products that only consider the indirect CFE on satellite reflectance, but not the direct CFE on gas exchange [i.e., FluxCom, MOD-C55, MOD-C6, and vegetation photosynthesis model (VPM)], do not report an increasing GPP trend (Fig. 3*B*). Their GPP trends are not a proxy of CFE because they model GPP solely based on climate forcings and leaf greenness or its surrogates. The climate forcings in reanalysis data may show negative impacts on GPP because of excessive temperature and drought stress in the tropics (see the EBF column in *SI Appendix*, Table S3), and the weak trends in leaf greenness cannot fully extrapolate the direct CFE effect because of the saturation of reflectance in EBFs (56–62). While the choice of climate forcing could introduce some uncertainties to our CFE estimates, we find similar diagnostic results with another forcing dataset [i.e., Climatic Research Unit and Japanese 55-year Reanalysis (CRU-JRA55) in *SI Appendix*, Fig. S7]. Furthermore, we calculate ${\text{β}}_{\mathrm{app}}^{\text{ln}}$ and ${\text{β}}_{\mathrm{dir}}^{\text{ln}}$ for the global-scale analysis (*SI Appendix*, Table S3), which results in the same conclusions as those using the relative GPP trend metric.

## The Partial Differential Sensitivity of GPP to CO_2_.

**Fig. 4.**
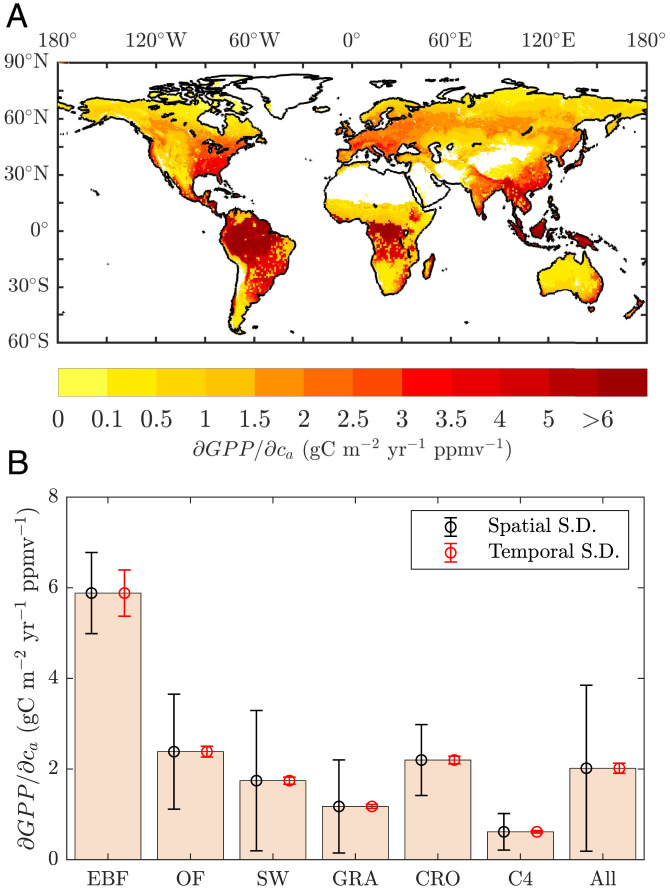
Average sensitivity of annual GPP to CO_2_ over 2001 to 2018. (*A*) Spatially distributed sensitivity of GPP to CO_2_. (*B*) Ecosystem-level sensitivity of GPP to CO_2_. Black error bars indicate 1 SD spatial variation. Red error bars indicate 1 SD temporal variation. The EEO framework is calibrated by the ensemble mean of eight satellite-derived GPP products.

To further characterize the underlying CFE, we show the spatial distribution of β_CO2_, which disentangles the confounding effects from other factors. The magnitude of β_CO2_ is largest in hot and humid regions at the annual scale (Fig. 4*A*). Since the CO_2_ trend is assumed to be constant worldwide, the spatial pattern of β_CO2_ represents that of CO_2_-induced GPP trends (A2 in *SI Appendix*, Fig. S5). At the ecosystem level, β_CO2_ is highest in EBFs at about 5.8 gC m^−2^ year^−1^ per ppmv CO_2_, followed by croplands and other forests from the temperate to boreal regions at about 2.2 gC m^−2^ year^−1^ per ppmv CO_2_ (Fig. 4*B*). EBFs have the longest growing season, and their high temperature and radiation determine the high photosynthetic capacity (*V_cmax_* and *J_max_*) and thus β_CO2_ (4, 28, 35). On the other hand, croplands cultivated in mild regions also have high photosynthetic capacity (63) and additional resource inputs from human management, including multiple cropping and irrigation (51), leading to a relatively high annual β_CO2_. Compared with C3 plants, our results suggest a positive but much weaker β_CO2_ in C4 plants (3, 4, 13), on the order of 0.6 gC m^−2^ year^−1^ per ppmv CO_2_, because the C4 CO_2_ pump inhibits the benefit of CFE (49).

According to plant photosynthesis optimization theories and gauged by the GPP magnitude estimated from an EC network or satellites, our EEO framework analytically constrains the sensitivity of GPP to CO_2_ (i.e., β_CO2_) from the gas exchange perspective. Our results suggest that the analytical solution of β_CO2_ is insensitive to the GPP uncertainty used for calibration of the framework (Fig. 1*A*; *SI Appendix*, Table S2) and largely unaffected by interannual fluctuations of climatic conditions in recent years (Fig. 4*B*). This highlights the advantage of the EEO framework in that the partial derivatives decouple the confounding effects of climate on CFE. A recent study using regression methods showed a 40% decline in CFE from 1982 to 2015 (16). However, their key findings are subject to the large temporal uncertainty in the satellite data and limited by the causal ambiguity of their regression (64–66). In addition, our EEO framework assumes that plants coordinate nutrient investment to optimize photosynthetic capacity (*V_cmax_* and *J_max_*) with environmental constraints (34–37), rather than prescribing specific values for plant functional types. Photosynthetic capacity not only controls the slope of the GPP response to CO_2_ but also strongly influences it by determining whether photosynthesis is light saturated or light limited (27, 28). We explore the influence of coordination at different timescales, comparing the results with the EC-inferred GPP (Fig. 1*A*; *SI Appendix*, Fig. S8), and adopt a decadal coordination to the environments in the peak LAI month for nutrient investment (67, 68) while adjusting the enzyme kinetics monthly with temperature (38). These assumptions result in a fixed reference *J_max_*/*V_cmax_* ratio for each site (or grid) over the study period while still allowing both to vary spatially to account for different growing climates. The decadal coordination allows for the presence of light-saturated and light-limited photosynthesis because the coordination is set at a much longer timescale than the stomatal and leaf *c_i_* optimization (35). Under these conditions, the EEO framework diagnoses a large proportion of marginally light-saturated photosynthesis, which responds more strongly to changes in *c_a_* than does light-limited photosynthesis (27, 28). Specifically, 63 to 69% of C3 canopy photosynthesis originates from light-saturated conditions for all seasons using the ERA5-land forcing. Together, these features explain the high β_CO2_ predicted by our framework and also lead to a good agreement with the overall trend in EC-inferred GPP and supported by multiple independent studies (39–42).

## Conclusion

We detect a large increase in GPP from a globally distributed EC network. Our EEO framework successfully captures the trends and IAVs in the EC-inferred GPP. The site-scale analysis suggests that CO_2_ is the dominant contributor to the GPP trends, while SWC and specific humidity control the IAVs. Using this framework to scale the GPP globally, the estimated absolute CO_2_-caused GPP trend is comparable to its EC-inferred counterpart and translates this CO_2_ fertilization effect to a global increase in photosynthesis of 4.1% decade^−1^ since the 2000s (or 5.1 PgC decade^−2^ for global sum, 4.4 gC m^−2^ year^−2^ for global average). However, our EEO framework diagnoses a high CFE compared with the median values of the DGVMs and the satellite-derived GPP products, with the largest disagreement in tropical forests. These tropical forests are responsible for about one-third of global GPP, and thus, an accurate estimation of CFE in tropical forests is critical to global carbon cycle modeling. Finally, we urge expansion of the currently sparse EC observing network in tropical ecosystems to improve understanding of their physiological responses to CO_2_ and climate change.

## Materials and Methods

### EEO Framework for GPP and CFE.

We derive a parsimonious EEO framework that constrains plant photosynthesis and water loss at a monthly scale. In addition to attributing changes in GPP through factorial simulations, the framework allows for partial differential sensitivity of GPP to atmospheric CO_2_ and various measurable environmental conditions. This is particularly important as neither GPP nor CFE can be measured directly in the natural environment, and the apparent changes in GPP are due to the confounding effect of changes in CO_2_ and other environmental conditions. We use Fick’s law and FvCB photosynthesis model as the governing equations for leaf-level photosynthesis. Photosynthesis is then constrained by three well-established optimization theories, which are widely supported by theoretical and empirical evidence (3, 4, 30, 32, 34–37, 44, 48, 69–73). Reconciling these optimization theories aims to constrain key variables in photosynthesis (33), such as the marginal water-use efficiency (mWUE) (74), stomatal conductance (*g*), leaf intercellular CO_2_ concentrations (*c_i_*), and photosynthetic capacity. Details of the EEO framework are derived in the SI Appendix, Text S1 to S7. We briefly summarize the concept of this framework below.

According to Fick’s law of mass transfer, neglecting the leaf boundary layer and mesophyll resistances, leaf-level photosynthesis is the product of stomatal conductance (*g*) and the gradient between atmospheric (*c_a_*) and leaf intercellular CO_2_ concentrations (*c_i_*). For a first intuitive guess, an increase in *c_a_* can lead to increased photosynthesis. However, plants actively optimize the tradeoff between carbon gain and water loss. Optimization partially offsets the direct photosynthetic benefits of elevated *c_a_* by indirectly reducing *g* and increasing *c_i_* to increase the carbon gain per unit water loss eventually.

• Theory 1 states that vegetation maximizes the sum of carbon gain and water loss through optimizing *g* in response to changing environment (30).
• Theory 2 states that vegetation minimizes the summed cost of transpiration and carboxylation per unit carbon assimilation through optimizing *c_i_* (32).

Specifically, theory 1 constrains *g*, and theory 2 constrains *c_i_*. Combining theories 1 and 2 provides a direct constraint on the mWUE. The optimization of *g* and *c_i_* should be sufficient and necessary because not only can the variation in *g* affect *c_i_* by controlling the inflow rate of ambient CO_2_ (30), but also the variation in *c_i_* can regulate stomatal aperture by adjusting guard cell membrane potential (4, 75, 76). To further consider the water stress on photosynthesis, we use a logistic function to approximate the change of mWUE with SWC (*SI Appendix*, Fig. S9) (33, 49, 72, 77). This directly allows the framework to incorporate the impact of SWC into the photosynthetic optimization processes (*SI Appendix*, Fig. S10) by calibrating a site-specific constant (i.e., ζ*_o_*; *SI Appendix*, Text S4).

In terms of the biogeochemical response, we assume that plants optimize nutrient allocation in response to the elevated CO_2_ and their growing environment. We use the photosynthetic coordination theory (theory 3) to constrain how plants optimize their nutrient investment, which allows for the estimation of photosynthetic capacity (i.e., *V_cmax_* and *J_max_*) without prescribing biome types (34–37).

• Theory 3 states that at a longer timescale than theories 1 and 2, plants adjust reference photosynthetic capacity to a nearly equal limitation of photosynthesis under average daytime conditions by ribulose-1,5-bisphosphate carboxylase (Rubisco) activity and ribulose-1,5-bisphosphate (RuBP) regeneration (interchangeable with light saturated and light limited, respectively) (34–37).

In other words, the coordination theory states that leaf nutrient content is regulated to reflect photosynthetic capacity, which is opposite to the hypothesis that photosynthetic capacity is limited by leaf nutrient content (78). Although the theory is well supported by multiple studies (34–37), determining the causation between the photosynthetic capacity and leaf nutrient content, or indeed a third alternative of finding common factors that limit the two, warrants further investigation (78). Nevertheless, the coordination theory captures the correlation between photosynthetic capacity and leaf nutrient content and implies that nutrient limitation should be implicit in LAI (37). We tested different timescales of the photosynthetic capacity coordination with respect to the monthly optimization of *g* and *c_i_* and compared our results with the EC-derived GPP (*SI Appendix*, Fig. S8). We use the timescale that best captures the trend and IAV of the EC-derived GPP (Fig. 1*A*) as the basis of our analysis. That is, the reference rates of *V_cmax_* and *J_max_* acclimate to the average environment of the peak LAI month during the study period (67, 68). For the other months, *V_cmax_* and *J_max_* are a function of temperature and their reference values (38). Therefore, our framework allows the photosynthetic capacity to be optimized to the changing atmospheric CO_2_ and meteorological conditions on a decadal timescale, and the photosynthesis is roughly colimited by Rubisco and RuBP. Other timescales explored include reference *V_cmax_* and *J_max_* values optimized according to 1) year-to-year variation of peak month CO_2_ + average of peak month meteorological conditions during the study period, 2) year-to-year variation of peak month CO_2_ and meteorological conditions, 3) month-to-month variation of CO_2_ + average of peak month meteorological conditions during the study period, and 4) month-to-month variation of CO_2_ and meteorological conditions. These represent a range of acclimation timescales, from short-term monthly and annual acclimation to longer-term decadal acclimation.

In order to preserve the parsimonious and analytical nature of the EEO framework, leaf-level photosynthesis is upscaled by LAI using a big-leaf approach. Changes in LAI implicitly account for canopy-scale effects of growing season extensions, recovery from disturbance, and succession. The upscaling factor for light extinction, denoted as $\bar{|\frac{G\left(\text{μ}\right)}{\text{μ}}|}$, represents the monthly average canopy shape (*G*) and solar zenith angle (μ) of a particular location (SI Appendix, Text S5). This factor is obtained by calibration with the EC-inferred GPP for site-scale analysis and satellite-derived GPP for global-scale analysis. $\bar{|\frac{G\left(\text{μ}\right)}{\text{μ}}|}$ for each month is then kept constant without year-to-year variation. We stress that the statistical significance of annual trends and interannual variations in EEO-inferred GPP are not caused by calibration but by the seven forcing variables: ambient CO_2_ concentration (*c_a_*), LAI, air temperature (*T_a_*), volumetric SWC, specific humidity (*q_a_*), incident shortwave radiation (*SW_in_*), and surface pressure (*P*). We finally evaluate the EEO framework qualitatively, which meets six of the seven criteria proposed by Ref. 72 (*SI Appendix*, Text S6).

A key benefit of the analytical framework, in addition to allowing a direct estimate of GPP, is that it can be used to assess the partial derivatives of GPP to different variables, which can further be used to reconstruct a composite estimate of GPP changes for attribution analysis. The following equation can express the change in GPP and sensitivities of GPP to multiple factors:

[1] ΔGPP=∂GPP∂caΔca+∂GPP∂LAIΔLAI+∂GPP∂TaΔTa+∂GPP∂SWCΔSWC+∂GPP∂qaΔqa+∂GPP∂SWinΔSWin+∂GPP∂PΔP,

where Δ*GPP* is the GPP change; $\frac{\partial \mathrm{GPP}}{\partial c_{a}}\Delta c_{a}$ is the direct CFE; $\frac{\partial \mathrm{GPP}}{\partial c_{a}}$ is the partial derivative of GPP to CO_2_, which reflects the absolute response of GPP to unit change in CO_2_; and so on. We treat these variables as independent forcings to different physiological processes within the EEO framework, but these physiological processes eventually interact to influence the gas exchange. For those larger-scale land–atmosphere interactions, the covariances between the forcing variables are implicit in their measurements (or reanalysis data). We denote $\frac{\partial \mathrm{GPP}}{\partial c_{a}}$ as β_CO2_ for simplicity. The symbol Δ can either be the interannual variation or long-term trend. To decompose the sensitivity of GPP to *T_a_* into different pathways (i.e., Fig. 2*C*), we additionally use the chain rule of calculus, as follows.

[2] $$\frac{\partial \mathrm{GPP}}{\partial T_{a}}=\frac{\partial \mathrm{GPP}}{\partial D}\frac{\partial D}{\partial T_{a}}+\frac{\partial \mathrm{GPP}}{\partial V_{\mathrm{cmax}}}\frac{V_{\mathrm{cmax}}}{\partial T_{a}}+\frac{\partial \mathrm{GPP}}{\partial K}\frac{\partial K}{\partial T_{a}}+\frac{\partial \mathrm{GPP}}{\partial {\text{Γ}}^{*}}\frac{\partial {\text{Γ}}^{*}}{\partial T_{a}}+\frac{\partial \mathrm{GPP}}{\partial {\text{η}}^{*}}\frac{\partial {\text{η}}^{*}}{\partial T_{a}},$$

where *D* is VPD, *V_cmax_* should be replaced with *J_max_* if the photosynthesis is light limited, *K* is the Michaelis-Menten coefficient, Γ* is the CO_2_ compensation point, and η* is the ratio of water viscosity under ambient air temperature to a reference temperature at 298.15 K. The full analytical form of the partial derivatives can be retrieved from the MATLAB script in the *SI Appendix*.

To compare with other studies, we compute the changes in GPP relativized by changes in CO_2_ with a metric used in Ref. 8:

[3] $${\text{β}}^{\text{ln}}=\left(\ln\;\frac{GPP_{e}}{GPP_{s}}\right)/\left(\ln\;\frac{c_{a,e}}{c_{a,s}}\right),$$

where *GPP_s_* and *c_a_*_,_*_s_* are of values in the starting year and *GPP_e_* and *c_a_*_,_*_e_* are of values in the ending year. A β^ln^ value of a unity indicates direct proportionality GPP response to CO_2_. We remind that this β^ln^ factor is only for intercomparison purposes; it is not a partial derivative and does not, by default, isolate the response of GPP to factors other than CO_2_ as β_CO2_ does. Since our framework can analytically attribute the trends to individual factors, we distinguish direct CO_2_ responses, ${\text{β}}_{\mathrm{dir}}^{\text{ln}}$, from apparent "CO_2_ responses," ${\text{β}}_{\mathrm{app}}^{\text{ln}}$.

### FLUXNET Dataset.

In this study, site-scale EC-inferred GPP and meteorological forcings are provided by the FLUXNET2015 Tier 1 dataset, and the uncertainties associated with this dataset have been previously reviewed (14). To calculate the EEO-inferred GPP (and its sensitivities to various factors), we use air temperature “TA_F,” incoming shortwave radiation “SW_IN_F,” surface pressure “PA_F,” VPD “VPD_F” (convertible to specific humidity), and volumetric SWC “SWC_F_MDS_1” in the dataset as inputs. The EEO framework performs calculations on a monthly basis for daytime averages.

1. Filtering of meteorological data. We only use data in which air temperature, incoming shortwave radiation, and VPD are measured or gap filled with good quality [i.e., quality flag (QF) is 0 or 1]. The same filtering is also applied to the EC-inferred GPP (i.e., FLUXNET2015 GPP).
2. Calculation of monthly daytime averages. We first compute the monthly average of the variables at a given half-hour/hour and aggregate them to monthly daytime averages. Daytime is determined by the nighttime flag (QF = 0) provided by the dataset and further checked with the half-hourly/hourly shortwave radiation (>0 Wm^−2^).
3. Calculation of reference EC-inferred GPP. In order to compare our results with EC-inferred GPP, we use the mean of “GPP_NT_VUT_MEAN,” “GPP_DT_VUT_MEAN,” “GPP_NT_CUT_MEAN,” and “GPP_DT_CUT_MEAN” as the main reference of EC-inferred GPP. In addition, we also use the individual EC-inferred GPP based on different partitioning and friction velocity filtering methods to analyze the corresponding GPP uncertainty propagation (shaded area in Fig. 1*A*).
4. Exclusion of low-quality monthly data. We exclude all the monthly averages with less than 50% good-quality timestamps or negative GPP.
5. Aggregation of annual GPP. Since the quality filtering from steps 1 to 4 may lead to data gaps, in annual aggregations, we ensure that no sites have more than one missing value of EEO-inferred GPP and EC-inferred GPP during the growing season (defined as monthly climatological mean EC-inferred GPP > 30 gC m^−2^ mo^−1^). If there is one and only one such missing value during a year, EEO-inferred and EC-inferred GPP will be filled by their corresponding low-quality data without the filtering of step 4. Otherwise, that particular year will be excluded.
6. Calculation of GPP annual anomalies. We compute the annual anomalies of the EEO-inferred GPP and EC-inferred GPP for each site independently across the observational network (*SI Appendix*, Fig. S1) and aggregate the anomalies across sites (Fig. 1*A*).
7. Last, our evaluations are performed for those sites with more than 5 y of good-quality data at the annual scale.

After the preprocessing, there are 68 qualified flux sites (632 site-years) from 2001 to 2014, covering 10 different biome types, including croplands (10), closed shrublands (1), deciduous broadleaf forests (11), EBFs (4), evergreen needleleaf forests (18), grasslands (12), mixed forest (6), open shrublands (2), savannas (1), and woody savannas (3) (*SI Appendix*, Table S1). In the site-scale analysis, all biome types are treated as C3 species because of lack of information. The GPP trends of site-scale analysis are examined by the Mann-Kendall test (R package: https://cran.r-project.org/web/packages/zyp/index.html). An overall uncertainty of the site-scale GPP trends is characterized by the IQR of trends through resampling of site-years (Fig. 2*A*; *SI Appendix*, Fig. S3*D*). In addition, we calculated the IQRs due to site heterogeneity by resampling of sites (*SI Appendix*, Fig. S3 *B* and *E*) and due to the uneven distribution of sites and site-years over the study period by randomly shuffling the first-year data (*SI Appendix*, Fig. S3 *C* and *F*). The shaded area in Fig. 1*A* shows the variations in the EC-inferred GPP due to the partitioning and frictional velocity filtering methods and their propagation to the EEO-inferred GPP. On the other hand, we analyze the dataset without the quality filtering in steps 1, 2, and 4. This allows a comparison of our EEO-inferred GPP with the fully gap-filled FLUXNET data (*SI Appendix*, Fig. S3*A*), ensuring that the resulting findings are not due to data filtering.

### Global Meteorological Forcing Datasets.

Our main global-scale results are computed using the ERA5-land hourly data (2001 to 2018, 0.1° × 0.1°, https://cds.climate.copernicus.eu/cdsapp#!/dataset/10.24381/cds.e2161bac). We use the following variables: “2m temperature,” “surface solar radiation downward,” “surface pressure,” “2m dew point temperature” (converted to specific humidity), and “volumetric soil water layers 1 and 2” (0 to 28 cm). We convert them to a monthly daytime average by examining the downward surface solar radiation (>20 W m^−2^) and resampling to 0.5° × 0.5°.

We estimate another EEO-inferred GPP using CRU-JRA55 forcing (2001 to 2018, 0.5° × 0.5°, https://catalogue.ceda.ac.uk/uuid/13f3635174794bb98cf8ac4b0ee8f4ed) for comparison. We use the following variables: “temperature at 2m,” “maximum temperature at 2m,” “downward solar radiation flux,” “pressure,” and “specific humidity.” Since there is no volumetric SWC in CRU-JRA55, we adopt it from ERA5-land. Daytime temperature is averaged from “temperature at 2m” and “maximum temperature at 2m.”

### MODIS Leaf Area Index.

We use Collection 6 MODIS LAI as our LAI inputs (52). We refine the MODIS Terra and Aqua LAI (8-d frequency, 500 m) by checking their quality flags following a previous study (51). The quality of this dataset has been extensively validated (55) and reported elsewhere (51, 79). We convert the original 8-d LAI into monthly frequencies. For the flux-site analysis, we match the 500-m LAI to each site’s longitude and latitude and average over in a 3 × 3 window (1.5 km × 1.5 km). For the global-scale analysis, we convert the 500-m LAI into 0.5° × 0.5°.

### Atmospheric CO_2._

We use the monthly average atmospheric CO_2_ concentration from the Mauna Loa Observatory and the South Pole Observatory provided by NOAA's Earth System Research Laboratory (https://www.esrl.noaa.gov/gmd/ccgg/trends/).

### DGVM Gross Primary Production.

DGVM outputs from the Trends in Net Land-Atmosphere Exchange (TRENDY-v6) project are used in this study (http://dgvm.ceh.ac.uk/node/9) (1). Here, we use two model experiments from TRENDY-v6, either varying CO_2_ only (time-invariant “preindustrial” climate and land use mask, S1) or varying CO_2_, climate, and land use (all forcing time-varying, S3). We exclude Sheffield DGVM (SDGVM) as it has an unrealistic sudden drop in GPP after 2007. More details are documented in *SI Appendix*, Table S4.

### Satellite-Derived GPP Products.

The following GPP products are used: 1) Boreal Ecosystem Productivity Simulator (80), 2) Breathing Earth System Simulator (81), 3) FluxCom (82), 4) and 5) MODIS Collection 5.5 (MOD-C55) and Collection 6 (MOD-C6) (83), 6) photosynthesis model (Pmodel-s0) (44), 7) photosynthesis–respiration model (PRmodel) (7), and 8) VPM (84). The ensemble global mean GPP of all these eight products through 2001 to 2016 is about 119 PgC y^−1^. More details about these products are documented in *SI Appendix*, Table S4.

### Land Cover Maps.

In order to analyze the spatial pattern of our diagnosed results, we use Collection 6 MODIS yearly product as the reference land cover map (2001 to 2018, 0.05° × 0.05°) (85). We refine the yearly maps for the study period into a single map and resample to 0.5° × 0.5° by taking the mode class of each grid cell. We aggregate the International Geosphere-Biosphere Program classification types into five C3 biomes (EBFs, other forests, short woody vegetation, grasslands, croplands) and one C4 biome (*SI Appendix*, Fig. S11). There is no biome aggregation for EBFs. Other forests are aggregated from deciduous broadleaf forests, mixed forests, evergreen needleleaf forests, deciduous needleleaf forests, and woody savannas. Short woody vegetation includes closed shrublands, open shrublands, savannas, and permanent wetlands. Croplands include croplands and cropland/natural vegetation mosaics. Finally, grids with greater than 50% C4 vegetation are labeled as C4 according to the ISLSCP II C4 vegetation percentage (https://daac.ornl.gov/cgi-bin/dsviewer.pl?ds_id=932).

## Supplementary Material

Supplementary File

## Data Availability

The following data were used in this work: FLUXNET2015: https://fluxnet.org/data/fluxnet2015-dataset/; MODIS LAI: https://lpdaac.usgs.gov/products/mod15a2hv006/ and https://lpdaac.usgs.gov/products/myd15a2hv006/; ERA5-land: https://cds.climate.copernicus.eu/cdsapp#!/dataset/10.24381/cds.e2161bac; CRU-JRA55: https://catalogue.ceda.ac.uk/uuid/13f3635174794bb98cf8ac4b0ee8f4ed; TRENDY-v6: http://dgvm.ceh.ac.uk/node/9/; MODIS land cover: https://lpdaac.usgs.gov/products/mcd12c1v006/; ISLSCP II C4 vegetation percentage: https://daac.ornl.gov/ISLSCP_II/guides/c4_percent_1deg.html; NOAA Atmospheric CO_2_: https://www.esrl.noaa.gov/gmd/ccgg/trends/; BESS GPP: https://www.environment.snu.ac.kr/bess-flux; MOD-C55 GPP: https://www.ntsg.umt.edu/project/modis/mod17.php; MOD-C6 GPP: https://lpdaac.usgs.gov/products/mod17a2hv006/; FluxCom GPP: http://www.fluxcom.org/CF-Products/; Pmodel-s0: https://zenodo.org/record/1423484#.YgMTS_XMIow; VPM GPP: https://figshare.com/articles/dataset/Monthly_GPP_at_0_5_degree/5048011. All other study data are referenced in the article. Codes are included in the supporting information.
