# Supplementary material for: CO2 fertilization of terrestrial photosynthesis inferred from site to global scales
Source: Proc Natl Acad Sci U S A. 2022 Mar 1;119(10):e2115627119. doi: 10.1073/pnas.2115627119 (PMC8915860; doi:10.1073/pnas.2115627119)
Supplement: Supplementary File [file pnas.2115627119.sapp.pdf]

**Supporting Information for**

# $CO_2$ fertilization of terrestrial photosynthesis inferred from site to global scales

Chi Chen[a,b,1], William J. Riley[a], I. Colin Prentice[c], Trevor F. Keenan[a,b,1]

[a]Climate and Ecosystem Sciences Division, Lawrence Berkeley National Laboratory, Berkeley, CA 94720, USA

[b]Department of Environmental Science, Policy and Management, UC Berkeley, Berkeley, CA 94720, USA

[c]Department of Life Sciences, Imperial College London, Ascot, SL5 7PY, UK

[1]To whom correspondence may be addressed. Email: chenchi@lbl.gov or trevorkeenan@berkeley.edu

**This PDF file includes:**

Text S1 to S7

Figs. S1 to S12

Tables S1 to S4

SI References

**S1. Leaf-level photosynthesis**

Fick's law of mass transfer describes the exchange of $CO_2$ and water vapor fluxes between the leaf and the atmosphere. Neglecting the leaf boundary layer and mesophyll resistances,

$$f_c = g(c_a - c_i) \tag{S1.1}$$

$$f_e = 1.6g(e_i - e_a) \approx 1.6gD \tag{S1.2}$$

where $f_c$ is the $CO_2$ flux [μmol m$^2$ s$^{-1}$], $f_e$ is the water vapor flux [mol m$^2$ s$^{-1}$], $g$ is the stomatal conductance for $CO_2$ [mol m$^2$ s$^{-1}$], $c_a$ and $c_i$ are ambient and intercellular $CO_2$ concentration [μmol mol$^{-1}$], respectively, 1.6 is the unitless relative diffusivity of water vapor with respect to $CO_2$, $e_a$ and $e_i$ are the ambient and intercellular water vapor concentration [mol mol$^{-1}$], respectively, and $D$ is the vapour pressure deficit [mol mol$^{-1}$] which is a proxy of $e_i - e_a$ when the leaf and atmosphere are well coupled (1). In our attribution analysis, we decompose $D$ into the air temperature ($T_a$) and specific humidity ($q_a$) using $D = e_a^* - e_a = \frac{611}{P}\exp\left(\frac{17.27(273.15-T_a)}{T_a-273.15+237.3}\right) - \frac{q_a}{0.622})$ (2).

The $CO_2$ flux can also be described using the Farquhar biochemical model of photosynthesis (3), which describes photosynthesis under light-saturated and -limited conditions:

$$\text{Light-saturated: } f_c = \frac{V_{cmax}(n_1 c_i - n_2\Gamma^*)}{K + n_1 c_i} - R_d \tag{S2.1}$$

$$\text{Light-limited: } f_c = \frac{J(n_1 c_i - n_2\Gamma^*)}{4(n_1 c_i + 2n_2\Gamma^*)} - R_d \tag{S2.2}$$

where $V_{cmax}$ is the maximum rate of Ribulose-1,5-bisphosphate carboxylase-oxygenase (Rubisco) activity [μmol m$^2$ s$^{-1}$], $K$ is the Michaelis-Menten coefficient [μmol mol$^{-1}$] (Eq. A1) for photosynthesis at an $O_2$ concentration of 209.5 mol mol$^{-1}$, $\Gamma^*$ is the $CO_2$ compensation point [μmol mol$^{-1}$] in the absence of dark respiration (Eq. A2), $R_d = 0.015V_{cmax}$ is the dark respiration rate [μmol m$^2$ s$^{-1}$], $J$ is the rate of electron transport [μmol m$^2$ s$^{-1}$]; Eq. S2.1 represents the light-saturated condition when Rubisco activity limits photosynthesis, while Eq. S2.2 represents the light-limited condition when Ribulose-1,5-bisphosphate (RuBP) regeneration limits photosynthesis. For consistency, we apply the same biochemical model to both C3 and C4 species, with differing parameters $n_1$ and $n_2$. For C3 species, both $n_1$ and $n_2$ are set to 1; for C4 species, $n_1$=25 (i.e., $CO_2$ pump) (4–6) and $n_2 = 0$ (zero $CO_2$ compensation point) (4, 7).

*S1.1. Optimality under light-saturated conditions*

In this section, we derive key variables for photosynthesis under light-saturated conditions. These key variables are $c_i$, $g$, and marginal water use efficiency ($\lambda$), with which we can constrain photosynthesis at the leaf-scale. Following refs. (1, 8), we linearize the Farquhar model (Eq. S2.1), assuming that $\Gamma^* \ll c_i$ , $R_d$ is sufficiently small, and that changes in $c_i$ have a minimal effect on the denominator of Eq. S2.1. The latter assumption leads to the approximation of $K + c_i$ to $K + sc_a$, where $s$ is treated as a constant equal to the long-term average of $\frac{c_i}{c_a}$. We stress that $c_i$ (or $s$) is a constant only in the denominator of Eq. S2 (1, 8). Thus, Eq. S2.1 can be rewritten as

$$f_c = \frac{V_{cmax} n_1 c_i}{K + n_1 s c_a} \tag{S3}$$

Combining Eqs. S1.1 and S3, we have

$$\frac{c_i}{c_a} = \frac{K + n_1 s c_a}{\frac{n_1 V_{cmax}}{g} + K + n_1 s c_a} \tag{S4.1}$$

$$f_c = \frac{g n_1 V_{cmax} c_a}{n_1 V_{cmax} + g(K + n_1 s c_a)} \tag{S4.2}$$

According to Theory #1, the objective function is a function of $g$ that

$$f_g(g) = f_c - \lambda f_e = \frac{g n_1 V_{cmax} c_a}{n_1 V_{cmax} + g(K + n_1 s c_a)} - 1.6\lambda g D \tag{S5}$$

where $\lambda$ is the marginal water use efficiency (mWUE) [μmol $CO_2$ mol$^{-1}$ $H_2O$], which depicts the gain of carbon flux per unit of water vapor loss. We assume $\lambda$ is a constant with respect to $g$ during a given time period (1, 8, 9). The maximum of the objective function can be obtained by finding $g$ when:

$$\frac{\partial f_g(g)}{\partial g} = \frac{(n_1 V_{cmax})^2 c_a}{[n_1 V_{cmax} + g(K + n_1 s c_a)]^2} - 1.6D = 0 \tag{S6}$$

We assume that $\frac{\partial \lambda}{\partial g} = 0$ or its contribution can be negligible (1, 8). Solving for $g$ in Eq. S6 yields:

$$g = \frac{n_1 V_{cmax}}{K + n_1 s c_a}\left[-1 + \left(\frac{c_a}{1.6\lambda D}\right)^{\frac{1}{2}}\right] \tag{S7}$$

Replacing Eq. S7 back into Eq. S4.1 and rearranging, we have

$$c_i = c_a\left[1 - \left(\frac{1.6\lambda D}{c_a}\right)^{\frac{1}{2}}\right] \tag{S8}$$

The unknown variable $\lambda$ is empirically reported to be proportional to $c_a$ (1, 4, 8, 10–12). To constrain this variable, we introduce a second optimization theory (i.e., the least-cost theory) (13), which is also widely supported by empirical evidence (14–18). Theory #2 states that under well-watered conditions, plants minimize the cost function

$$f_{c_i}(c_i) = a\frac{f_e}{f_c} + b\frac{V_{cmax}}{f_c} \tag{S9}$$

where $a$ is the carbon cost of maintaining the transpiration required to support assimilation at an assimilation rate ($f_c$) and $b$ is the cost of maintaining photosynthetic proteins at the level required to support at the same assimilation rate (13). Practically, $b$ is treated as the ratio of total 24-hour leaf maintenance respiration to $V_{cmax}$, and $a$ is taken as the ratio of leaf maintenance respiration cost of the sapwood ($R_s$) to transpiration ($f_e$) (13). The ratio $\frac{b}{a}$ is further expressed as

$$\frac{b}{a} = \frac{b}{a'\eta^*} = \frac{146 \times 10^{-6}}{\eta^*} \tag{S10}$$

where $\frac{b}{a'}$ is found to be 146×10$^{-6}$ [mol μmol$^{-1}$] according to the global leaf δ$^{13}$C dataset (18), $\eta^*$ is the ratio of water viscosity at ambient temperature to a reference temperature at 298.15 K, which can be expressed by Vogel's equation (16, 19) (Eq. A6). By setting $\frac{\partial f_{c_i}(c_i)}{\partial c_i} = 0$, one arrives at

$$\frac{\partial f_{c_i}(c_i)}{\partial c_i} = a\frac{\partial (f_e/f_c)}{\partial c_i} + b\frac{\partial (V_{cmax}/f_c)}{\partial c_i} = 0 \tag{S11}$$

To solve this equation, first, we assume that $a$ and $b$ are independent of $c_i$. Second, we note that Theory #1 and #2 can hold simultaneously. That is, the variation in $g$ can affect $c_i$ (1), but also the variation in $c_i$ can simultaneously regulate stomatal aperture by adjusting guard cell membrane potential through changing ion and organic solute concentrations (20–22). Third, the purpose of Eq. S11 is to find the optimal $c_i$, which is slightly different from finding the optimal $\frac{c_i}{c_a}$ as in ref. (13) (they become an equivalent problem if $c_a$ is a constant during a short period, e.g., within a month). Nevertheless, we note that our solutions for $c_i$ and $\frac{c_i}{c_a}$ (Eq. S16) are identical to ref. (13). Connecting Eqs. S1.1 and S1.2, we get

$$\frac{f_e}{f_c} = \frac{1.6D}{c_a - c_i} \tag{S12}$$

Rewriting Eq. S2.1 by neglecting $\Gamma^*$ and $R_d$, one arrives

$$f_c = \frac{n_1 V_{cmax} c_i}{K + n_1 c_i} \tag{S13}$$

Here, $c_i$ is the variable to be optimized. Thus, $c_i$ in the denominator of Eq. S13 can no longer be treated as a constant (like in Eq. S3). Accordingly,

$$\frac{V_{cmax}}{f_c} = \frac{n_1 c_i}{K + n_1 c_i} \tag{S14}$$

Further connecting Eqs. S11, S12, and S14 results in

$$\frac{\partial f_{c_i}(c_i)}{\partial c_i} = a\frac{1.6D}{(c_a - c_i)^2} - b\frac{K}{n_1 {c_i}^2} = 0 \tag{S15}$$

Solving the quadratic Eq. S15 for $c_i$ yields

$$c_i = c_a \frac{1}{1 + \left(1.6\frac{aDn_1}{bK}\right)^{\frac{1}{2}}} \tag{S16}$$

The other root for $c_i$ is discarded because the ratio $\frac{c_i}{c_a}$ must be less than 1. Connecting Eqs. S8 and S16, we get the solution for $\lambda$:

$$\lambda = c_a \frac{an_1}{bK} \frac{1}{\left[1 + \left(1.6\frac{aDn_1}{bK}\right)^{\frac{1}{2}}\right]^2} \tag{S17}$$

Eq. S17 shows that $\lambda$ will increase proportionally with increasing $c_a$, and it becomes more water-efficient under a lower vapor pressure deficit. We note that the response of $\lambda$ to soil water content is further described in Text S4.

*S1.2. Optimality under light-limited conditions*

In order to derive $c_i$ and $g$ under light-limited conditions, we linearized Eq. S2.2 of the Farquhar model, similar to the derivation under light-saturation conditions. In other words, we assume that $\Gamma^* \ll c_i$ in the numerator (this is supported by Theory #3 as $c_i$ should be maintained at a high level, similar to under light-saturated conditions), $R_d$ is sufficiently small with respect to $f_c$, and that the variability of $c_i$ has a minimal effect on the denominator of Eq. S2.2, which results in

$$f_c = \frac{Jn_1 c_i}{4(n_1 s c_a + 2n_2\Gamma^*)} \tag{S18}$$

Connecting Eq. S1.1 and Eq. S18, we have

$$\frac{c_i}{c_a} = \frac{4g(n_1 s c_a + 2n_2\Gamma^*)}{4g(n_1 s c_a + 2n_2\Gamma^*) + n_1 J} \quad \text{(S19.1)}$$

$$f_c = \frac{n_1 g J c_a}{4g(n_1 s c_a + 2n_2\Gamma^*) + n_1 J} \quad \text{(S19.2)}$$

The objective function is:

$$f_g(g) = f_c - \lambda f_e = \frac{n_1 g J c_a}{4g(n_1 s c_a + 2n_2\Gamma^*) + n_1 J} - 1.6\lambda g D \quad \text{(S20)}$$

By setting $\frac{\partial f_g(g)}{\partial g} = 0$, we have

$$\frac{\partial f_g(g)}{\partial g} = \frac{(n_1 J)^2 c_a}{[4g(n_1 s c_a + 2n_2\Gamma^*) + n_1 J]^2} - 1.6\lambda D = 0 \quad \text{(S21)}$$

Solving for $g$ arrives

$$g = \frac{n_1 J}{4(n_1 s c_a + 2n_2\Gamma^*)}\left[-1 + \left(\frac{c_a}{1.6\lambda D}\right)^{\frac{1}{2}}\right] \quad \text{(S22)}$$

where $\lambda$ is independent of the light condition and equal to that described in Eq. S17. The expression for $g$ under light-limited conditions is similar to that under light-saturated conditions (Eq. S7). Importantly, inserting Eq. S22 into Eq. S19 yields the same expression for $c_i$ as derived under light-saturated conditions (i.e., Eq. S8). Indeed, this is a necessary condition to derive $V_{cmax}$ and $J_{max}$ (the maximum rate of electron transport [mol$^{-1}$ mol]) using the photosynthesis coordination theory (i.e., Rubisco activity and RuBP regeneration co-limit the photosynthesis, Text S2) (23, 24).

## S2. Calculation of the photosynthetic capacity

Empirical evidence supports that the vegetation photosynthetic capacity acclimates to the environment, including radiation, temperature, ambient $CO_2$ concentration (16, 18, 25–28). The photosynthesis coordination theory (Theory #3) provides the theoretical basis for predicting the response of photosynthetic capacity $V_{cmax}$ and $J_{max}$ to the environment, which does not require information on plant functional types (with the exception being C3/C4 species). The theory states that Rubisco activity and RuBP regeneration co-limit photosynthesis under average growing environments (14–16, 18, 23, 24, 29). By exploring the timescale of photosynthetic capacity coordination (see Materials and Methods), we assume that the reference values of $V_{cmax}$ and $J_{max}$ acclimate to the average environment of the peak LAI month during the study period. For the rest

of the months, $V_{cmax}$ and $J_{max}$ are a function of temperature and their reference values (25). The coordination of $V_{cmax}$ and $J_{max}$ at longer timescale (i.e., decadal) than the optimization of stomatal conductance and leaf $c_i$ (i.e., monthly in this study) allows for the presence of light-saturated and light-limited photosynthesis. We briefly describe the derivation of reference photosynthetic capacity, $V_{cmax}^{ref}$ and $J_{max}^{ref}$, and the details can be found in ref. (23). Note that all input variables for Eqs. S24-29 are the average environment of the peak LAI month during the study period unless otherwise noted.

$J$ is a saturation function of incident radiation and converges to $J_{max}$:

$$\theta J^2 - (\varphi PPFD + J_{max})J + \varphi PPFD J_{max} = 0 \tag{S24}$$

$$PPFD = 4.55 SW_{in} f_{PAR}$$

$J$ is then taken as the smaller root of Eq. S24:

$$J = \varphi PPFD + J_{max} - \sqrt{(\varphi PPFD + J_{max})^2 - 4\theta\varphi PPFD J_{max}} \tag{S25}$$

where $\varphi = 0.085$ is the quantum yield of photosynthetic electron transport [mol mol$^{-1}$], $PPFD$ is the photosynthetic photon flux density [µmol], $f_{PAR} = 0.45$ is the fraction of photosynthetically active radiation (PAR) in the incident shortwave radiation $SW_{in}$, and 4.55 [µmol J$^{-1}$] is the conversion ratio of PAR-energy, $\theta = 0.85$ is the curvature of the light response curve (23, 24). To derive the optimal $J_{max}$, we assume that the light-limited $f_c$ changes proportionally with $J_{max}$ (23, 24, 30):

$$\frac{\partial f_c}{\partial J_{max}} = c \tag{S26}$$

where $c = 0.053$ (23, 24). Further connecting Eqs. S2.2, S25, and S26 and neglecting dark respiration, $J_{max}^{ref}$ is solved as follows.

$$J_{max}^{ref} = \varphi PPFD \varpi \tag{S27}$$

$$\varpi = -(1 - 2\theta) + \sqrt{(1-\theta)\left(\frac{1}{\frac{4c}{m}\left(1 - \theta\frac{4c}{m}\right)} - 4\theta\right)}$$

$$m = \frac{n_1 c_i - n_2\Gamma^*}{n_1 c_{i+} 2n_2\Gamma^*}$$

To calculate $V_{cmax}$, the Rubisco activity and RuBP regeneration co-limiting theory equates Eqs. S2.1 and S2.2:

$$\frac{V_{cmax}(c_i - \Gamma^*)}{K + c_i} = \frac{J(c_i - \Gamma^*)}{4(c_{i+} 2\Gamma^*)} \quad \text{(S28)}$$

where $c_i$ is shown to be independent of light conditions (Text S1.2). For simplicity, we further denote $m_c = \frac{n_1 c_i - n_2 \Gamma^*}{K + n_1 c_i}$. Incorporating Eqs.S25, S27, and S28, we term $V_{cmax}^{ref}$:

$$V_{cmax}^{ref} = \varphi PPFD \left(\frac{m}{m_c}\right)\left(\frac{\varpi^*}{8\theta}\right) \quad \text{(S29)}$$

$$\varpi^* = 1 + \varpi - \sqrt{(1+\varpi)^2 - 4\theta\varpi}$$

We emphasize that in our study, plants coordinate the allocation of resources for photosynthetic capacity during the climatological peak LAI month with the corresponding climatological mean environmental conditions. For the other months, $V_{cmax}$ and $J_{cmax}$ are adjusted using the temperature acclimation function (25):

$$V_{cmax} = V_{cmax}^{ref} \exp\left[\frac{\Delta H_v\left(T - T_{ref}\right)}{T_{ref} R T}\right] \frac{1 + \exp\left[\frac{T_{ref}\Delta S_v - H_d}{T_{ref} R}\right]}{1 + \exp\left[\frac{T\Delta S_v - H_d}{TR}\right]} \quad \text{(S30)}$$

$$\Delta S_v = a_{s,v} - b_{s,v}(T - 273.15)$$

where $T$ is the temperature of the interested month [K], $\Delta H_v$ is the activation energy for [71,513 J mol$^{-1}$], $H_d$ is the deactivation energy [200,000 J mol$^{-1}$], $T_{ref}$ is the climatology air temperature corresponding to the climatology peak LAI month [K], $R$ is the universal gas constant [8.3145 J mol$^{-1}$ K$^{-1}$], $\Delta S_v$ is an entropy term [J mol$^{-1}$ K$^{-1}$], $a_{s,v}$ is the intercept term [668.39 J mol$^{-1}$ K$^{-1}$], and $b_{s,v}$ is the slope term [1.07 J mol$^{-1}$ K$^{-1}$]. Similar to $V_{cmax}$,

$$J_{max} = J_{max}^{ref} \exp\left[\frac{\Delta H_J\left(T - T_{ref}\right)}{T_{ref} R T}\right] \frac{1 + \exp\left[\frac{T_{ref}\Delta S_J - H_d}{T_{ref} R}\right]}{1 + \exp\left[\frac{T\Delta S_J - H_d}{TR}\right]} \quad \text{(S31)}$$

$$\Delta S_j = a_{s,J} - b_{s,J}(T - 273.15)$$

where $\Delta H_J$ is activation energy for [49,884 J mol$^{-1}$], $\Delta S_j$ is an entropy term [J mol$^{-1}$ K$^{-1}$], $a_{s,J}$ is the intercept term [659.7 J mol$^{-1}$ K$^{-1}$], and $b_{s,J}$ is the slope term [0.75 J mol$^{-1}$ K$^{-1}$] (25). The final expression for $J$ is obtained by inserting $J_{max}$ of Eq. S31 into Eq. S25 with inputs from the month of interest.

## S3. Canopy upscaling

The above derivations relate to leaf-scale photosynthesis. The leaf-scale quantum yield of photosynthetic electron transport ($\varphi = 0.257$ mol mol$^{-1}$) describes the efficiency with which PAR energy is converted to fixed carbon per unit leaf area for a single interaction with photons. In reality, there are multiple scatterings and absorption of photons in the canopy, which requires canopy upscaling. We upscale leaf-scale, $\varphi$, to the effective canopy-scale, $\varphi_c$, using a big-leaf model approach:

$$\varphi_c = \varphi \times \left[ \frac{1 - \exp\left( - \overline{\left| \frac{G(\mu)}{\mu} \right|} \times LAI \right)}{\overline{\left| \frac{G(\mu)}{\mu} \right|}} \right] \tag{S32}$$

where $LAI$ is leaf area index, and $\overline{\left| \frac{G(\mu)}{\mu} \right|}$ is a lumped factor that accounts for the average canopy geometry shape and solar zenith angle, and $\mu$ is the cosine of solar zenith angle. For each month, we assume that there is no year-to-year variation in monthly $\overline{\left| \frac{G(\mu)}{\mu} \right|}$. Therefore, after calibrating with known carbon flux, $\overline{\left| \frac{G(\mu)}{\mu} \right|}$ is set to a monthly constant for a particular location. To calibrate $\overline{\left| \frac{G(\mu)}{\mu} \right|}$, we use the least squares method to find the optimal $\overline{\left| \frac{G(\mu)}{\mu} \right|}$ by comparing EEO-inferred GPP with EC-inferred GPP (i.e., FLUXNET2015 GPP) for the site-scale analysis, or with the average of 8 satellite-derived GPP products for the global-scale analysis. $\varphi_c$ is then used to compute the canopy-scale stomatal conductance and photosynthesis. The use of the inverse parameter $\overline{\left| \frac{G(\mu)}{\mu} \right|}$ provides a bulk estimate of the effective big-leaf area for canopy photosynthesis, and thus does not require additional information to explicitly model the radiation field.

## S4. Effect of soil water content on optimization and mWUE

The above derivations are in the case of adequate soil water content ($SWC$). However, the optimality of stomatal conductance (or carbon-water coupling) can also be strongly influenced by plant water hydraulics under drought stress (4, 12, 18, 31–37). Eqs. S1 and S2 only describe the coupling of carbon and water diffusion between the plant and the atmosphere, while the linkage of soil-plant interaction on photosynthesis is absent. In order to account for the effect of $SWC$ on

carbon-water coupling and to maintain the continuum in soil–plant–atmosphere interaction, we introduce a modified logistic function imposing soil moisture stress on mWUE ($\lambda$), which directly couples the optimization of stomatal conductance and leaf intercellular $CO_2$ concentration (4, 32, 33), modifying Eq.S17 as follows.

$$\lambda' = F(\zeta_o, SWC)\lambda = \frac{1+\exp(-\zeta_o SWC)}{1-\exp(-\zeta_o SWC)} c_a \frac{an_1}{bK} \frac{1}{\left[1+\left(1.6\frac{aDn_1}{bK}\right)^{\frac{1}{2}}\right]^2} \tag{S33}$$

where $\lambda'$ is the marginal water use efficiency after accounting for the soil moisture effect, $SWC$ is the volumetric soil water content [$m^3$ $m^{-3}$], and $\zeta_o$ is a site-specific parameter that needs to be calibrated with either the EC-inferred measurements (i.e., FLUXNET2015 GPP) or the ensemble mean of multiple GPP products using the least squares method. The soil water stress imposed on $\lambda$ by $F(\zeta_o, SWC)$ can be passed to the ratio $\frac{a}{b}$ (which also contains information on the soil-plant water interaction) in the least-cost theory (Eq. S10) by connecting Eqs. S8 and S16. We illustrate the characteristics of $\lambda'$ and SWC in Fig. S9 and Fig. S10. Given a fixed $\zeta_o$, under wet conditions (high $SWC$), $F(\zeta_o, SWC)$ is close to unity and has little effect on $\lambda'$. Under dry conditions (low $SWC$), $\lambda'$ will increase to enable a conservative water-use, leading to a reduction in the stomatal conductance and photosynthesis. Under extremely dry conditions, $F(\zeta_o, SWC)$ and $\lambda'$ will increase sharply, and the plant will dramatically decrease the stomatal conductance and photosynthesis. $\zeta_o$ characterizes the underlying water-use strategy of a plant. When $\zeta_o$ is low, the plant is more isohydric and the stomatal conductance is sensitive to $SWC$.

**S5. The final EEO framework for GPP**

The final form of the canopy-scale GPP [μmol $m^2$ $s^{-1}$] takes the smaller value of Eq. S2. Among the important variables, $V_{cmax}$ and $J_{max}$ are expressed by Eqs. S30 and S31 by replacing $\varphi$ with $\varphi_c$ (Eq. S32). $c_i$ is described in Eq. S8 by replacing $\lambda$ with $\lambda'$ (Eq. S33). $\Gamma^*$, $K$, $R_d$ are described in SI Appendix text S7. In addition, there are two free parameters in this framework, $\left|\frac{\overline{G(\mu)}}{\mu}\right|$ and $\zeta_o$. We iteratively use the method of least squares to calibrate these two parameters in our framework to the EC-inferred GPP (i.e., FLUXNET2015 GPP) for the site-scale analysis, or to the average of 8 satellite-derived GPP products for the global-scale analysis. The maximum number of iterations

is 50, or the iteration stops when changes in $\left|\frac{\overline{G(\mu)}}{\mu}\right|$<0.05 & $\zeta_o$<0.001 & $V_{cmax}$<5 μmol m$^{-2}$ s$^{-1}$ between two consecutive iterations, whichever comes first. The threshold value set for $V_{cmax}$ aims to speed up the convergence of iteration. For the first iteration, we guess a value of $\left|\frac{\overline{G(\mu)}}{\mu}\right|$ by assuming the plant is under well-watered condition (i.e., set the initial value of $\zeta_o$ to +∞). From the second interaction onwards, the least-square optimization of $\zeta_o$ kicks in and $V_{cmax}$ responds accordingly. Each $\left|\frac{\overline{G(\mu)}}{\mu}\right|$ and $\zeta_o$ are then held as constants. We stress that trends and IAVs in the EEO-inferred GPP are only caused by the seven forcing variables ($c_a$, LAI, $T_a$, SWC, $q_a$, $SW_{in}$ and $P$), but not by the calibration of the free parameters $\left|\frac{\overline{G(\mu)}}{\mu}\right|$ and $\zeta_o$. In other words, the calibration of $\left|\frac{\overline{G(\mu)}}{\mu}\right|$ and $\zeta_o$ do not influence the statistical significance in trend test. Fig. S12 shows the spatial patterns of the mean GPP estimated by our framework from 2001 to 2016 at the global scale using ERA5-land forcing and MODIS Collection 6 LAI, as well as the GPP from the 8 satellite-derived GPP products and their ensemble climatological means.

**S6. Qualitative evaluation of the optimization model**

We qualitatively evaluate the optimization model by seven criteria (32). Follow ref. (32) and based on Theory #1 (i.e., max ($f_c + \lambda' f_e$)), the water penalty is

$$\Theta=\lambda' f_e \quad \text{(S34)}$$

An equivalent water penalty could be obtained by Theory #2 as its solution is equivalent to Theory #1 (Eqs. S8 and S16). Therefore, the discriminant of the criteria is expressed as

$$\frac{\partial \Theta}{\partial f_e} = \frac{\partial f_c}{\partial f_e} = \lambda' \quad \text{(S35)}$$

where $\lambda'$ is shown in Eq. S33. Our framework does not violate six of the seven criteria in ref. (32):

- **C1: $\frac{\partial \Theta}{\partial f_e} > 0$ when $f_e > 0$.** Our $\frac{\partial \Theta}{\partial f_e}$ is always non-negative.
- **C2: The $\frac{\partial \Theta}{\partial f_e}$ is a nondecreasing function of $f_e$.** Due to the model assumption, the model has a unique solution for $\frac{\partial \Theta}{\partial f_e}$ which is not a function of $f_e$; thus, our framework does not violate this criterion.
- **C3: The $\frac{\partial \Theta}{\partial f_e} \leq \frac{\partial f_c}{\partial f_e}$ when $f_e = 0$.** Our $\frac{\partial \Theta}{\partial f_e} = \frac{\partial f_c}{\partial f_e}$.

- **C4: The $\frac{\partial\Theta}{\partial f_e}$ is a nonincreasing function of vapour pressure deficit for a given $f_e$.** Our $\frac{\partial\Theta}{\partial f_e}$ decreases as vapor pressure deficit (i.e., $D$) increases.
- **C5: The $\frac{\partial\Theta}{\partial f_e}$ is a nondecreasing function of $c_a$ for a given $f_e$.** Our $\frac{\partial\Theta}{\partial f_e}$ increases as $c_a$ increases.
- **C6: The $\frac{\partial\Theta}{\partial f_e}$ is a monotonically increasing function of soil drought for a given $f_e$.** Our $\frac{\partial\Theta}{\partial f_e}$ decreases as soil water content (SWC) decreases.
- **C7: The $\frac{\partial\Theta}{\partial f_e}$ is a monotonically increasing function of prior loss of hydraulic conductivity for a given $f_e$.** For each site or grid cell, our $\frac{\partial\Theta}{\partial f_e}$ does not respond to hydraulic conductivity.

**S7. Appendix**

The Michaelis-Menten coefficient $K$ is computed as follows.

$$K = K_c\left(1 + \frac{C_{oa}}{K_o}\right) \tag{A1}$$

$$K_c = K_{c,25}\frac{\exp\left[\Delta H_{K_c}(T - T_{0k})\right]}{TRT_{0k}}$$

$$K_o = K_{o,25}\frac{\exp\left[\Delta H_{K_o}(T - T_{0k})\right]}{TRT_{0k}}$$

where $K_c$ is the Michaelis-Menten constant for $CO_2$ [μmol mol$^{-1}$], $K_o$ is the Michaelis-Menten constant for carboxylation and oxygenation reaction [mmol mol$^{-1}$], $K_{c,25}$ is the Michaelis-Menten constant for $CO_2$ at 25°C [μmol mol$^{-1}$], $K_{o,25}$ is the Michaelis-Menten constant for carboxylation and oxygenation reaction 25°C [mmol mol$^{-1}$], $C_{oa}$ is the concentration for oxygen [209.5 mmol mol$^{-1}$], $\Delta H_{K_c}$is the activation energy for $K_c$ [79,430 J mol$^{-1}$], $\Delta H_{K_o}$ is the activation energy for $K_o$ [36,380 J mol$^{-1}$], $T_{0k}$ is 273.15K, and $R$ is the universal gas constant [8.3145 J mol$^{-1}$ K$^{-1}$]. These values are provided by ref. (38).

The $CO_2$ compensation point $\Gamma^*$ is calculated according to the Arrhenius equation (18, 38):

$$\Gamma^* = \Gamma^*{}_{25}\exp\left[\frac{\Delta H_{\Gamma^*}(T - T_{0k})}{TRT_{0k}}\right]\frac{P_s}{P_0} \tag{A2}$$

where $\Gamma^*{}_{25}$ is 42.75 μmol mol–1, $\Delta H_{\Gamma^*}$ is 37,830 J mol–1 (38), $P_s$ is the surface pressure [Pa], and $P_0$ is the standard atmospheric pressure at sea level [101,325 Pa].

$\eta^*$ is the ratio of water viscosity under ambient temperature to a reference temperature at 298.15 K. According to Vogel's equation (16, 19), water viscosity at a given ambient temperature $T$ can be expressed as:

$$\eta = 10^{-3}\exp\left(A + \frac{B}{C+T}\right) \tag{A3}$$

where $A = -3.719$, $B = 580$, $C = -138$. Thus, the sensitivity of $\eta$ to temperature is

$$\frac{1}{\eta}\frac{\partial\eta}{\partial T} = \frac{\partial \ln\eta}{\partial T} = \frac{-B}{(C+T)^2} \tag{A4}$$

Therefore,

$$\ln\eta|_{298.15K}^{T} = \frac{B}{T+C}\bigg|_{298.15K}^{T} \tag{A5}$$

$$\eta^* = \frac{\eta}{\eta_{298.15K}} = \exp\left[\frac{-B(T-298.15)}{(T+C)(298.15+C)}\right] \tag{A6}$$

**Table S1.** Site information of the FLUXNET2015 data used in this study.

| | Valid years | | | | | | | | | | | | | | LAT | LON | IGBP | Study period |
|---|---|---|---|---|---|---|---|---|---|---|---|---|---|---|---|---|---|---|
| AT-Neu | ○ | ○ | ● | ● | ● | ● | ● | ● | ● | ● | ● | ● | ○ | ○ | 47.12 | 11.32 | GRA | 2002 - 2012 (10) |
| AU-DaP | ○ | ○ | ○ | ○ | ○ | ○ | ○ | ● | ● | ● | ● | ● | ○ | ○ | -14.06 | 131.32 | GRA | 2007 - 2013 (5) |
| AU-DaS | ○ | ○ | ○ | ○ | ○ | ○ | ○ | ● | ● | ● | ● | ● | ● | ● | -14.16 | 131.39 | SAV | 2008 - 2014 (7) |
| AU-How | ○ | ○ | ● | ○ | ● | ○ | ○ | ● | ○ | ● | ● | ● | ● | ● | -12.49 | 131.15 | WSA | 2001 - 2014 (8) |
| AU-Stp | ○ | ○ | ○ | ○ | ○ | ○ | ○ | ○ | ○ | ● | ● | ● | ● | ● | -17.15 | 133.35 | GRA | 2008 - 2014 (5) |
| AU-Tum | ○ | ● | ● | ● | ● | ● | ● | ● | ● | ● | ● | ○ | ● | ● | -35.66 | 148.15 | EBF | 2001 - 2014 (12) |
| BE-Lon | ○ | ○ | ○ | ● | ● | ● | ○ | ● | ● | ○ | ● | ● | ● | ● | 50.55 | 4.75 | CRO | 2004 - 2014 (9) |
| BE-Vie | ● | ● | ● | ● | ● | ● | ● | ● | ○ | ● | ● | ● | ● | ● | 50.30 | 6.00 | MF | 2001 - 2014 (13) |
| CA-Gro | ○ | ○ | ○ | ● | ● | ● | ● | ● | ● | ● | ● | ● | ● | ○ | 48.22 | -82.16 | MF | 2003 - 2014 (10) |
| CA-Oas | ● | ● | ● | ● | ● | ● | ● | ● | ● | ● | ○ | ○ | ○ | ○ | 53.63 | -106.20 | DBF | 2001 - 2010 (10) |
| CA-Qfo | ○ | ○ | ○ | ● | ● | ● | ● | ● | ● | ● | ○ | ○ | ○ | ○ | 49.69 | -74.34 | ENF | 2003 - 2010 (7) |
| CA-TP1 | ○ | ○ | ● | ● | ● | ● | ● | ○ | ● | ● | ● | ● | ● | ● | 42.66 | -80.56 | ENF | 2002 - 2014 (11) |
| CA-TP3 | ○ | ○ | ● | ○ | ● | ● | ● | ● | ○ | ● | ● | ● | ● | ● | 42.71 | -80.35 | ENF | 2002 - 2014 (10) |
| CA-TP4 | ○ | ● | ● | ● | ● | ● | ● | ● | ● | ● | ● | ● | ● | ● | 42.71 | -80.36 | ENF | 2002 - 2014 (13) |
| CH-Dav | ○ | ○ | ○ | ○ | ○ | ○ | ● | ● | ● | ● | ● | ● | ● | ● | 46.82 | 9.86 | ENF | 2001 - 2014 (8) |
| CH-Fru | ○ | ○ | ○ | ○ | ○ | ○ | ● | ● | ● | ● | ○ | ● | ● | ● | 47.12 | 8.54 | GRA | 2005 - 2014 (7) |
| CH-Lae | ○ | ○ | ○ | ○ | ● | ● | ● | ● | ● | ● | ● | ● | ● | ● | 47.48 | 8.36 | MF | 2004 - 2014 (10) |
| CH-Oe1 | ○ | ● | ● | ● | ● | ● | ● | ● | ○ | ○ | ○ | ○ | ○ | ○ | 47.29 | 7.73 | GRA | 2002 - 2008 (7) |
| CH-Oe2 | ○ | ○ | ○ | ○ | ○ | ○ | ○ | ○ | ● | ● | ● | ● | ● | ○ | 47.29 | 7.73 | CRO | 2004 - 2014 (5) |
| DE-Geb | ● | ● | ● | ● | ● | ● | ● | ● | ● | ● | ● | ● | ● | ● | 51.10 | 10.91 | CRO | 2001 - 2014 (14) |
| DE-Gri | ○ | ○ | ○ | ○ | ○ | ○ | ● | ● | ● | ● | ● | ● | ● | ● | 50.95 | 13.51 | GRA | 2004 - 2014 (8) |
| DE-Hai | ● | ● | ● | ● | ● | ● | ● | ● | ● | ● | ● | ● | ○ | ○ | 51.08 | 10.45 | DBF | 2001 - 2012 (12) |
| DE-Kli | ○ | ○ | ○ | ○ | ● | ● | ● | ● | ● | ● | ● | ● | ● | ● | 50.89 | 13.52 | CRO | 2004 - 2014 (10) |
| DE-Lnf | ○ | ● | ● | ● | ● | ● | ○ | ○ | ○ | ● | ● | ● | ○ | ○ | 51.33 | 10.37 | DBF | 2002 - 2012 (8) |
| DE-Obe | ○ | ○ | ○ | ○ | ○ | ○ | ○ | ○ | ○ | ● | ● | ● | ● | ● | 50.79 | 13.72 | ENF | 2008 - 2014 (5) |
| DE-Tha | ● | ● | ● | ● | ● | ● | ● | ● | ● | ● | ● | ● | ● | ● | 50.96 | 13.57 | ENF | 2001 - 2014 (14) |
| DK-Sor | ● | ● | ● | ● | ● | ● | ● | ● | ● | ● | ● | ● | ● | ● | 55.49 | 11.64 | DBF | 2001 - 2014 (14) |
| ES-LJu | ○ | ○ | ○ | ○ | ● | ● | ● | ● | ● | ● | ● | ● | ● | ○ | 36.93 | -2.75 | OSH | 2004 - 2013 (9) |
| FI-Hyy | ● | ● | ● | ● | ○ | ● | ● | ● | ● | ● | ○ | ● | ● | ○ | 61.85 | 24.29 | ENF | 2001 - 2014 (11) |
| FI-Sod | ● | ● | ● | ● | ● | ● | ● | ● | ● | ● | ● | ● | ● | ● | 67.36 | 26.64 | ENF | 2001 - 2014 (14) |
| FR-Gri | ○ | ○ | ○ | ○ | ○ | ● | ● | ● | ● | ● | ● | ○ | ○ | ○ | 48.84 | 1.95 | CRO | 2004 - 2014 (6) |
| GF-Guy | ○ | ○ | ○ | ● | ● | ● | ● | ● | ● | ● | ● | ● | ● | ● | 5.28 | -52.92 | EBF | 2004 - 2014 (11) |
| IT-BCi | ○ | ○ | ○ | ○ | ○ | ● | ● | ● | ● | ● | ○ | ○ | ○ | ○ | 40.52 | 14.96 | CRO | 2004 - 2014 (5) |
| IT-Cpz | ● | ○ | ○ | ● | ● | ● | ● | ● | ○ | ○ | ○ | ○ | ○ | ○ | 41.71 | 12.38 | EBF | 2001 - 2009 (6) |
| IT-Lav | ○ | ○ | ○ | ○ | ● | ● | ● | ● | ○ | ○ | ● | ○ | ● | ● | 45.96 | 11.28 | ENF | 2003 - 2014 (7) |
| IT-MBo | ○ | ○ | ○ | ○ | ● | ● | ● | ● | ● | ● | ● | ● | ● | ○ | 46.01 | 11.05 | GRA | 2003 - 2013 (9) |
| IT-Noe | ○ | ○ | ○ | ○ | ○ | ● | ● | ○ | ○ | ○ | ○ | ● | ● | ● | 40.61 | 8.15 | CSH | 2004 - 2014 (5) |
| IT-Ren | ○ | ● | ● | ○ | ● | ● | ● | ● | ● | ● | ● | ● | ● | ○ | 46.59 | 11.43 | ENF | 2001 - 2013 (11) |
| IT-Ro1 | ● | ○ | ● | ● | ● | ● | ○ | ● | ○ | ○ | ○ | ○ | ○ | ○ | 42.41 | 11.93 | DBF | 2001 - 2008 (6) |
| IT-Ro2 | ○ | ○ | ○ | ● | ● | ● | ● | ○ | ○ | ● | ● | ● | ○ | ○ | 42.39 | 11.92 | DBF | 2002 - 2012 (7) |
| IT-SRo | ○ | ○ | ● | ● | ● | ● | ● | ● | ● | ○ | ○ | ● | ○ | ○ | 43.73 | 10.28 | ENF | 2001 - 2012 (8) |
| IT-Tor | ○ | ○ | ○ | ○ | ○ | ○ | ○ | ○ | ● | ● | ● | ● | ● | ● | 45.84 | 7.58 | GRA | 2008 - 2014 (6) |
| MY-PSO | ○ | ○ | ● | ● | ● | ● | ● | ● | ● | ○ | ○ | ○ | ○ | ○ | 2.97 | 102.31 | EBF | 2003 - 2009 (7) |
| NL-Loo | ● | ● | ● | ● | ● | ● | ● | ● | ● | ● | ● | ● | ● | ● | 52.17 | 5.74 | ENF | 2001 - 2014 (14) |
| RU-Fyo | ○ | ○ | ○ | ○ | ○ | ● | ● | ● | ● | ● | ● | ● | ● | ● | 56.46 | 32.92 | ENF | 2001 - 2014 (9) |
| US-ARM | ○ | ○ | ● | ● | ● | ● | ● | ○ | ● | ● | ● | ○ | ○ | ○ | 36.61 | -97.49 | CRO | 2003 - 2012 (8) |
| US-Blo | ● | ● | ● | ● | ● | ● | ○ | ○ | ○ | ○ | ○ | ○ | ○ | ○ | 38.90 | -120.63 | ENF | 2001 - 2007 (6) |
| US-GLE | ○ | ○ | ○ | ○ | ● | ● | ● | ● | ● | ● | ● | ● | ● | ○ | 41.37 | -106.24 | ENF | 2004 - 2014 (9) |
| US-IB2 | ○ | ○ | ○ | ○ | ● | ● | ● | ● | ● | ● | ● | ○ | ○ | ○ | 41.84 | -88.24 | GRA | 2004 - 2011 (7) |
| US-MMS | ● | ● | ● | ● | ● | ● | ● | ● | ● | ● | ● | ● | ● | ● | 39.32 | -86.41 | DBF | 2001 - 2014 (14) |
| US-Me2 | ○ | ● | ● | ● | ● | ○ | ● | ● | ● | ● | ○ | ● | ● | ● | 44.45 | -121.56 | ENF | 2002 - 2014 (11) |
| US-NR1 | ○ | ● | ● | ● | ● | ● | ● | ● | ● | ● | ● | ● | ● | ● | 40.03 | -105.55 | ENF | 2001 - 2014 (13) |
| US-Ne1 | ○ | ● | ● | ● | ● | ● | ● | ● | ● | ● | ● | ● | ○ | ○ | 41.17 | -96.48 | CRO | 2001 - 2013 (11) |
| US-Ne2 | ○ | ● | ● | ● | ● | ● | ● | ● | ● | ● | ● | ● | ○ | ○ | 41.16 | -96.47 | CRO | 2001 - 2013 (11) |
| US-Ne3 | ○ | ● | ● | ● | ● | ● | ● | ● | ● | ● | ● | ● | ○ | ○ | 41.18 | -96.44 | CRO | 2001 - 2013 (11) |
| US-Oho | ○ | ○ | ○ | ● | ● | ● | ● | ● | ● | ● | ● | ● | ● | ○ | 41.55 | -83.84 | DBF | 2004 - 2013 (10) |
| US-PFa | ● | ● | ● | ● | ● | ● | ● | ● | ● | ● | ● | ● | ● | ● | 45.95 | -90.27 | MF | 2001 - 2014 (14) |
| US-SRC | ○ | ○ | ○ | ○ | ○ | ○ | ○ | ● | ● | ● | ● | ● | ○ | ○ | 31.91 | -110.84 | MF | 2008 - 2014 (5) |
| US-SRG | ○ | ○ | ○ | ○ | ○ | ○ | ○ | ● | ● | ● | ● | ● | ● | ● | 31.79 | -110.83 | GRA | 2008 - 2014 (7) |
| US-SRM | ○ | ○ | ○ | ● | ● | ● | ● | ● | ● | ● | ● | ● | ● | ● | 31.82 | -110.87 | WSA | 2004 - 2014 (11) |
| US-Syv | ○ | ● | ● | ● | ● | ● | ● | ○ | ○ | ○ | ○ | ○ | ○ | ● | 46.24 | -89.35 | MF | 2001 - 2014 (7) |
| US-Ton | ○ | ● | ● | ● | ● | ● | ● | ● | ● | ● | ● | ● | ● | ● | 38.43 | -120.97 | WSA | 2001 - 2014 (13) |
| US-UMB | ○ | ● | ● | ● | ● | ● | ● | ● | ● | ● | ● | ● | ● | ● | 45.56 | -84.71 | DBF | 2001 - 2014 (13) |
| US-UMd | ○ | ○ | ○ | ○ | ○ | ○ | ○ | ● | ● | ● | ● | ● | ● | ● | 45.56 | -84.70 | DBF | 2007 - 2014 (7) |
| US-Var | ● | ● | ● | ● | ● | ● | ● | ● | ● | ● | ● | ● | ● | ● | 38.41 | -120.95 | GRA | 2001 - 2014 (14) |
| US-WCr | ● | ● | ● | ● | ● | ● | ○ | ○ | ○ | ○ | ● | ● | ● | ● | 45.81 | -90.08 | DBF | 2001 - 2014 (10) |
| US-Whs | ○ | ○ | ○ | ○ | ○ | ○ | ○ | ● | ● | ● | ● | ● | ● | ● | 31.74 | -110.05 | OSH | 2007 - 2014 (7) |
| US-Wkg | ○ | ○ | ○ | ○ | ● | ● | ● | ● | ● | ● | ● | ● | ● | ● | 31.74 | -109.94 | GRA | 2004 - 2014 (10) |
| | | 2002 | | 2004 | | 2006 | | 2008 | | 2010 | | 2012 | | 2014 | | | | |

Solid circles indicate a valid year with qualified data. LON and LAT indicate the location of each site. IGBP is the biome type of each site. The numbers in paratheses in the column “Study period” show the number of valid years during our study period.

**Table S2.** $CO_2$-induced relative GPP trends diagnosed by the EEO framework (unit: % decade$^{-1}$).

| GPP source used to calibrate the EEO framework | EBF | OF | SW | GRA | CRO | C4 | All biomes |
|---|---|---|---|---|---|---|---|
| Ensemble mean of 8 satellite-derived GPP | 4.76 | 4.27 | 4.75 | 5.02 | 5.06 | 1.35 | 4.12 |
| BEPS | 4.89 | 4.50 | 4.85 | 5.18 | 5.35 | 1.41 | 4.36 |
| BESS | 4.85 | 4.29 | 4.76 | 5.16 | 5.14 | 1.38 | 4.24 |
| FluxCom | 4.81 | 4.35 | 4.77 | 4.85 | 4.91 | 1.28 | 4.07 |
| MOD-C55 | 4.76 | 4.37 | 4.88 | 5.19 | 5.12 | 1.37 | 4.20 |
| MOD-C6 | 4.69 | 4.36 | 4.89 | 5.22 | 5.11 | 1.38 | 4.17 |
| Pmodel-s0 | 4.71 | 4.06 | 4.52 | 4.66 | 4.71 | 1.25 | 3.91 |
| PR-model | 4.96 | 4.07 | 4.60 | 4.76 | 4.95 | 1.34 | 4.08 |
| VPM | 4.67 | 4.31 | 4.88 | 5.23 | 5.08 | 1.34 | 4.03 |

The row with "Ensemble mean of 8 satellite-derived GPP" represents bar "A2" in Fig. 3. Trends are divided by their respective mean EEO-inferred GPP from 2001 to 2016. As the $CO_2$ trend is assumed to be constant globally, this table also reflects the differences in sensitivity of GPP to $CO_2$ (i.e., $\beta_{CO2}$) among the different GPP products used for calibration.

**Table S3.** Logarithmic response ratio of changes in GPP with respect to changes in $CO_2$ at the global scale, i.e., $\beta^{\ln}$, during 2001-2016.

| | | EBF | OF | SW | CRO | GRA | C4 | All |
|---|---|---|---|---|---|---|---|---|
| EEO-inferred | Direct ($\beta_{app}^{\ln}$) | 0.71 | 1.02 | 1.01 | 0.88 | 1.18 | 0.42 | 0.81 |
| | Diagnosed full ($\beta_{app}^{\ln}$) | 0.71 | 1.10 | 1.09 | 0.86 | 1.15 | 0.44 | 0.84 |
| | Diagnosed $CO_2$ only ($\beta_{dir}^{\ln}$) | 0.84 | 0.76 | 0.85 | 0.88 | 0.91 | 0.24 | 0.73 |
| | Diagnosed LAI and Climate only | -0.13 | 0.36 | 0.22 | -0.02 | 0.26 | 0.20 | 0.10 |
| Satellite-derived ($\beta_{app}^{\ln}$) | BEPS | 0.49 | 1.08 | 1.10 | 1.42 | 1.67 | 0.80 | 0.92 |
| | BESS | 0.34 | 0.86 | 0.99 | 1.36 | 1.46 | 0.57 | 0.81 |
| | FluxCom | -0.08 | 0.03 | 0.00 | 0.06 | 0.06 | -0.02 | -0.01 |
| | MOD-C55 | -0.11 | 0.13 | 0.11 | 0.42 | 0.43 | 0.29 | 0.15 |
| | MOD-C6 | -0.04 | 0.73 | 1.13 | 1.36 | 1.02 | 1.03 | 0.68 |
| | Pmodel-s0 | 0.21 | 0.64 | 0.71 | 0.82 | 1.01 | 0.44 | 0.56 |
| | PRmodel | 0.11 | 0.37 | 0.64 | 0.76 | 0.79 | 0.28 | 0.44 |
| | VPM | -0.39 | 1.09 | 0.88 | 1.14 | 1.21 | 0.12 | 0.54 |
| | Median of multiple products | 0.04 | 0.64 | 0.71 | 0.82 | 1.01 | 0.44 | 0.54 |
| TRENDY-v6 full ($\beta_{app}^{\ln}$) | CABLE | 0.72 | 0.97 | 0.84 | 0.81 | 1.38 | 0.94 | 0.90 |
| | ISAM | 0.60 | 0.61 | 0.52 | 0.52 | 0.45 | 0.39 | 0.46 |
| | LPJ-GUESS | 0.31 | 0.77 | 0.74 | 0.89 | 0.94 | 0.35 | 0.61 |
| | LPJ-wsl | 0.22 | 0.60 | 0.46 | 0.81 | 0.78 | 0.35 | 0.53 |
| | VISIT | 0.40 | 1.04 | 0.83 | 1.01 | 0.92 | 0.44 | 0.78 |
| | CLASS-CTEM | 0.40 | 0.44 | 0.15 | -0.26 | 0.25 | -0.56 | 0.12 |
| | CLM4.5 | 0.55 | 0.59 | 0.48 | 0.38 | 0.67 | 0.23 | 0.52 |
| | DLEM | 0.61 | 1.14 | 0.90 | 1.12 | 1.14 | 0.82 | 0.86 |
| | JULES | -0.04 | 0.74 | 0.69 | 0.76 | 1.06 | 1.00 | 0.72 |
| | ORCHIDEE | 0.28 | 0.64 | 0.54 | 0.79 | 0.75 | 0.23 | 0.50 |
| | VEGAS | 0.19 | 0.53 | 0.25 | 0.18 | 0.68 | -0.24 | 0.24 |
| | JSBACH | 0.50 | 0.66 | 0.43 | 0.70 | 0.87 | -0.02 | 0.45 |
| | LPX-Bern | 0.14 | 0.41 | 0.42 | 0.62 | 0.35 | 0.18 | 0.32 |
| | Median of TRENDY-v6 full | 0.40 | 0.64 | 0.52 | 0.76 | 0.78 | 0.35 | 0.52 |
| TRENDY-v6 $CO_2$ only ($\beta_{dir}^{\ln}$) | CABLE | 0.79 | 0.60 | 0.81 | 1.08 | 0.60 | 1.54 | 0.86 |
| | ISAM | 0.69 | 0.56 | 0.57 | 0.68 | 0.83 | 0.59 | 0.65 |
| | LPJ-GUESS | 0.44 | 0.42 | 0.41 | 0.19 | 0.76 | 0.31 | 0.42 |
| | LPJ-wsl | 0.36 | 0.22 | 0.27 | 0.42 | 1.21 | 0.15 | 0.34 |
| | VISIT | 0.42 | 0.47 | 0.34 | 0.39 | 1.26 | 0.10 | 0.38 |
| | CLASS-CTEM | 0.47 | 0.81 | 0.34 | 0.58 | 1.83 | 0.44 | 0.62 |
| | CLM4.5 | 0.29 | 0.37 | 0.12 | 0.29 | 1.15 | 0.09 | 0.30 |
| | DLEM | 0.49 | 0.67 | 0.72 | 0.73 | 0.98 | 0.83 | 0.69 |
| | JULES | 1.13 | 0.70 | 0.56 | 0.96 | 1.99 | 0.32 | 0.81 |
| | ORCHIDEE | 0.54 | 0.50 | 0.43 | 0.44 | 0.54 | 0.38 | 0.51 |
| | VEGAS | 0.27 | 0.21 | 0.28 | 0.21 | 0.32 | 0.45 | 0.32 |
| | JSBACH | 0.46 | 0.64 | 0.48 | 0.82 | 1.65 | 0.66 | 0.67 |
| | LPX-Bern | 0.39 | 0.39 | 0.47 | 0.34 | -0.18 | 0.39 | 0.32 |
| | Median of TRENDY-v6 $CO_2$ only | 0.46 | 0.50 | 0.43 | 0.44 | 0.98 | 0.39 | 0.51 |

These response ratios are computed using Eq. 2 (or Eq. 1 of ref. (39)). The row called "LAI and Climate only" has no corresponding name for $\beta^{\ln}$, but still provides a response ratio for comparison use.

**Table S4.** Information for satellite-derived GPP products and Dynamic Global Vegetation Models used in this study.

| | Short name | Long name and other notes | Reference |
|---|---|---|---|
| **Satellite-derived GPP products** | BEPS | The Boreal Ecosystem Productivity Simulator | Ref. (40) |
| | BESS | The Breathing Earth System Simulator | Ref. (41) |
| | FluxCom | FluxCom RS+METEO, no explicit CFE | Ref. (42) |
| | MOD-C55 | Terra MODIS MOD17A2 Collection 5.5; no explicit CFE | Ref. (43) |
| | MOD-C6 | Terra MODIS MOD17A2HGF (gap filled) Collection 6; no explicit CFE | Ref. (43) |
| | Pmodel-s0 | Photosynthesis model S0 (empirical soil moisture stress) | Ref. (31) |
| | PRmodel | Photosynthesis–Respiration model | Ref. (17) |
| | VPM | Vegetation Photosynthesis Model; no explicit CFE | Ref. (44) |
| **TRENDY-v6 Dynamic Global Vegetation Models** | CABLE | The Community Atmosphere–Biosphere Land Exchange model | Ref. (45) |
| | ISAM | The Integrated Science Assessment Model | |
| | LPJ-GUESS | Lund-Potsdam-Jena General Ecosystem Simulator | |
| | LPJ-wsl | Lund–Potsdam–Jena Wald Schnee und Landscaft Version | |
| | VISIT | Vegetation Integrative SImulator for Trace gases | |
| | CLASS-CTEM | The Canadian Land Surface Scheme and the Canadian Terrestrial Ecosystem Model | |
| | CLM4.5 | The Community Land Model Version 4.5 | |
| | DLEM | Dynamic Land Ecosystem Model | |
| | JULES | The Joint UK Land Environment Simulator | |
| | ORCHIDEE | Organising Carbon and Hydrology In Dynamic Ecosystems; An alternative simulation ORCHIDEE-MICT is excluded as it does not turn on dynamic vegetation | |
| | VEGAS | VEgetation Global Atmosphere and Soil | |
| | JSBACH | Jena Scheme for Biosphere-Atmosphere Coupling in Hamburg; GPP is averaged from two types of simulations with and without pastures as pastures | |
| | LPX-Bern | Land surface Processes and eXchanges model of the University of Bern; GPP is averaged from two types of simulations with different land transition settings | |

All data were converted to a spatial resolution of 0.5°×0.5° and a monthly temporal resolution before analysis.

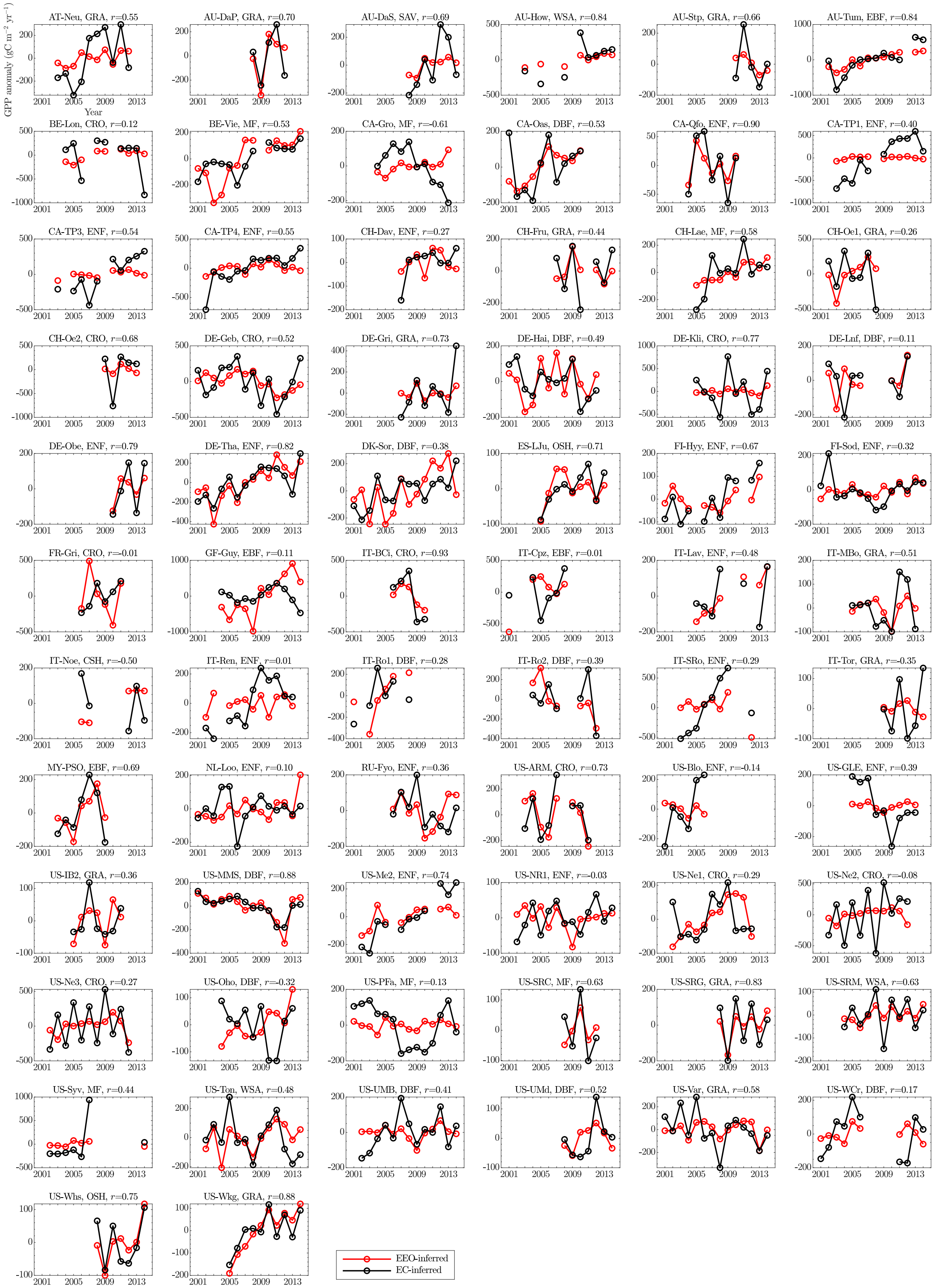


**Fig. S1.** Interannual variation of EEO-inferred GPP and EC-inferred GPP. Shown are 68 sites with more than 5 years good-quality data (see the 7 filtering steps in Materials and Methods).

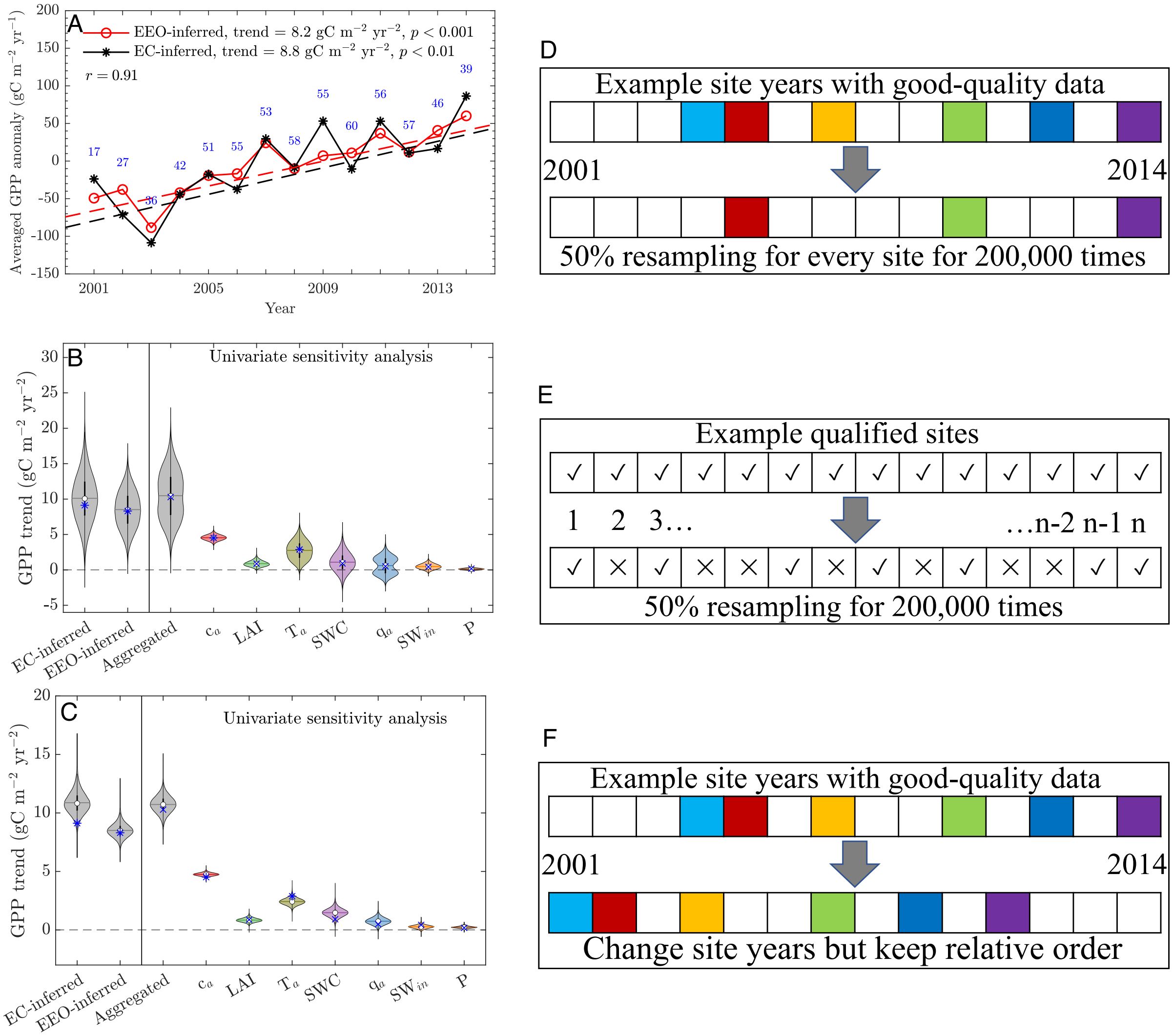


**Fig. S2.** Robustness of the framework to uncertainties. (A) Similar to Fig. 1A, trends and interannual variations of GPP without quality filtering. (B) Probability density of GPP trends showing uncertainty due to site heterogeneity. (C) Probability density of GPP trends showing uncertainty due to uneven distribution of sites and site-years over the study period. For B and C, each probability density consists of 200,000 resamples, realized by the "randperm" function in MATLAB. (D) Schematic of resampling site years for Fig. 2A. (E) Schematic of resampling site numbers for Fig. S3B. *n* is 68, i.e., the total number of the study sites. (F) Schematic of moving the first year of data for each site, with the relative order of the subsequent data kept constant, for Fig. S3C.

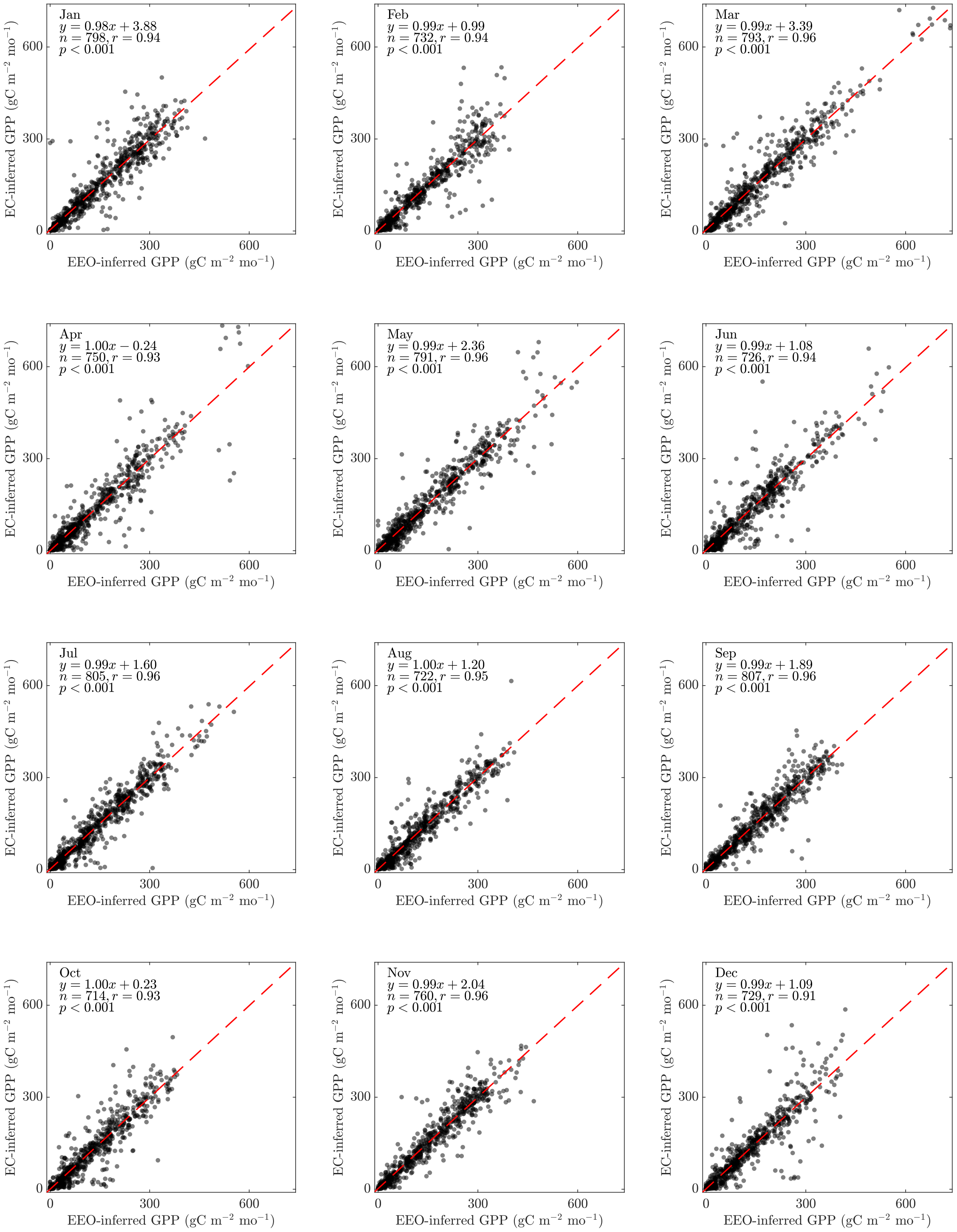


**Fig. S3.** Scatter plots of EEO-inferred and EC-inferred GPP at the monthly scale. Red lines indicate best-fit lines from linear regression.

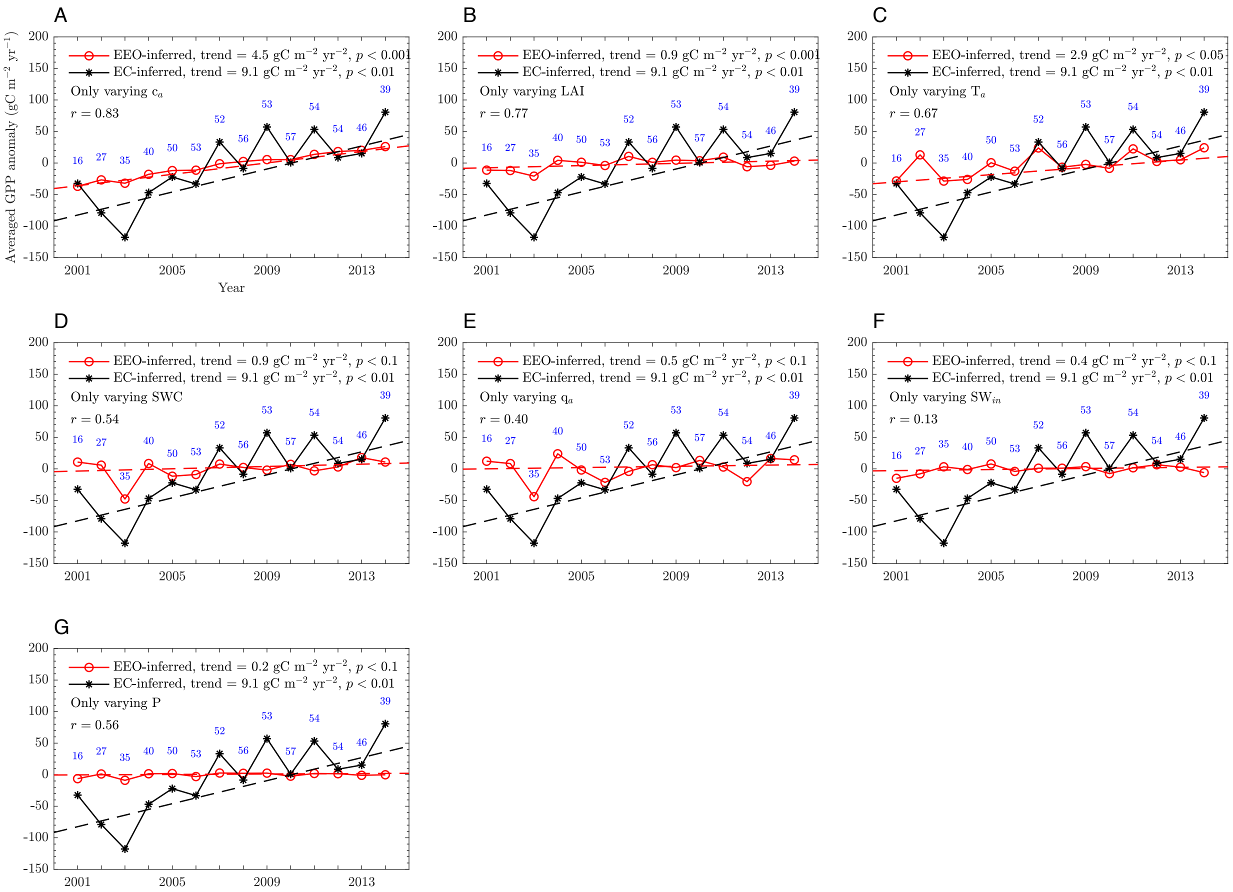


**Fig. S4.** GPP trends and year-to-year variations obtained by univariate sensitivity analysis using the EEO framework. The other variables are kept as the climatological mean of the study period. * indicates a statistically significant trend ($p<0.05$).

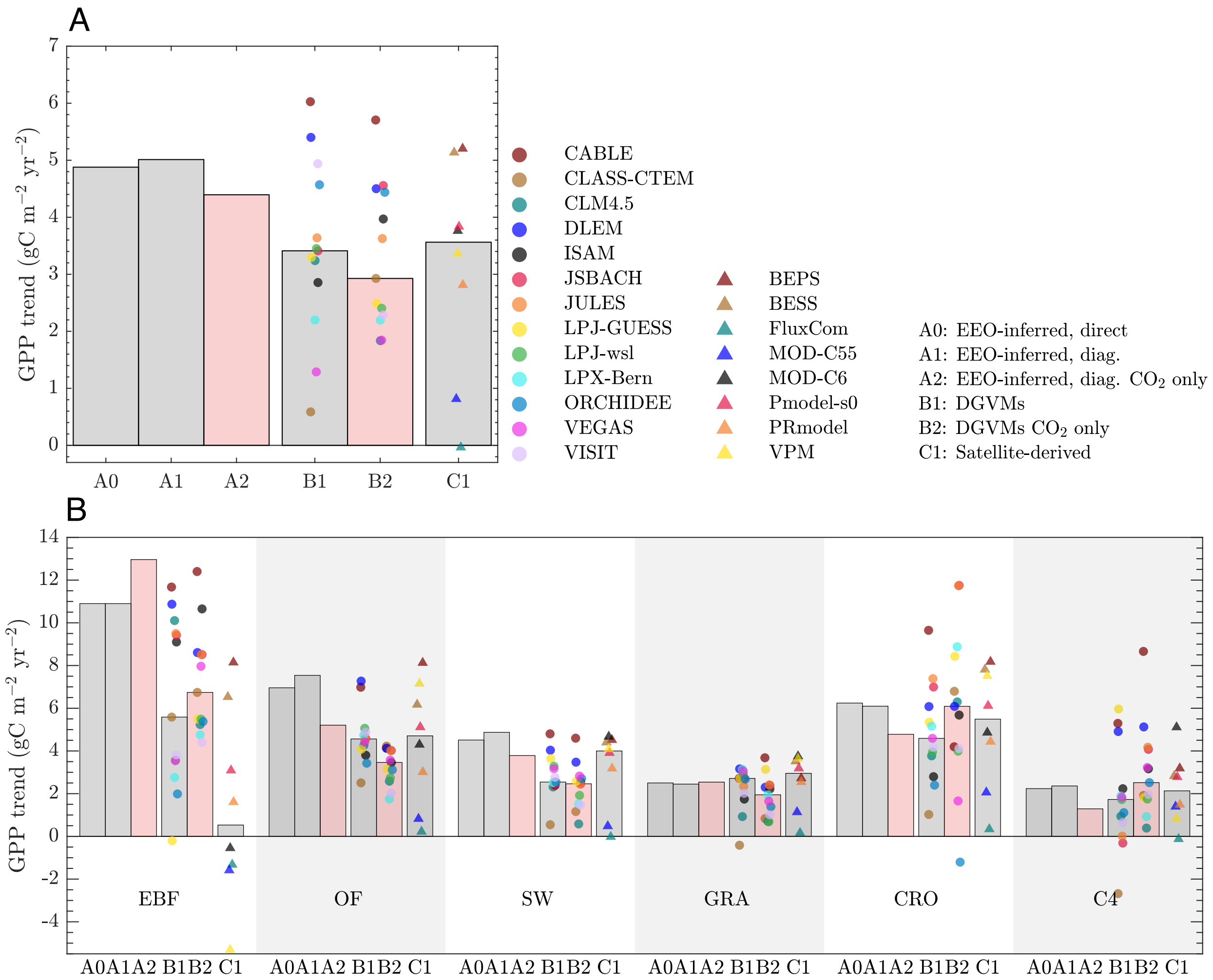


**Fig. S5.** Absolute GPP trends at the global scale from 2001 to 2016. (A) all biomes. (B) individual biomes, including evergreen broadleaf forests (EBF), other forests (OF), short woody vegetation (SW), grasslands (GRA), croplands (CRO), and C4 vegetation (C4). “A0”: estimated directly from our EEO framework. “A1”: diagnostic trends composited from partial derivatives using the EEO framework. “A2”: diagnostic $CO_2$-induced trends through the partial derivative approach using the EEO framework. “B1”: 13 DGVM models, all forcing time-varying. “B2”: 13 DGVM models, $CO_2$ only. “C1”: 8 satellite-derived GPP products. For B1, B2, and C1, the bars represent their median. The grey bars represent GPP trends considering all effects. The red bars represent GPP trends caused by $CO_2$ only.

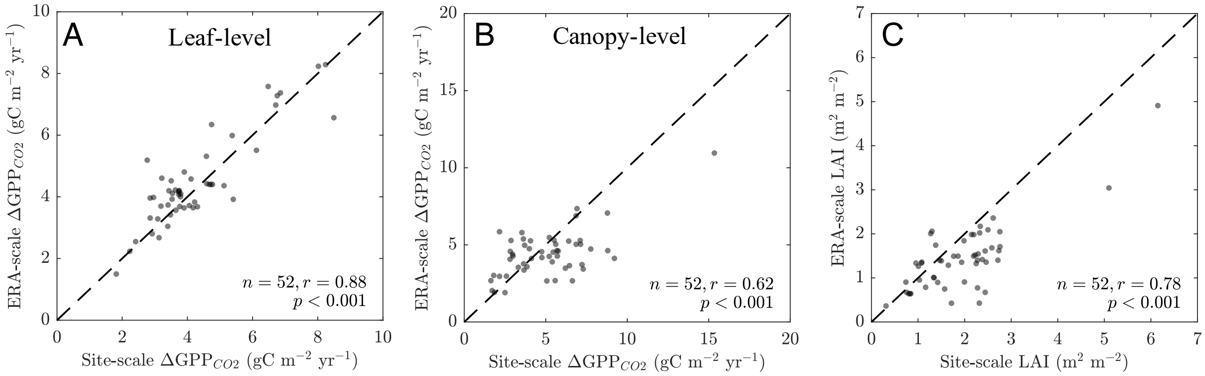


**Fig. S6**. Scatter plots of EEO results at different spatial scales. (A) Leaf-level comparison for $CO_2$-induced GPP trends at site-scale vs. ERA-scale (0.5°×0.5°). (B) Canopy-level comparison for $CO_2$-induced GPP trends at site-scale vs. ERA-scale. (C) LAI comparison at site-scale vs. ERA-scale (for LAI, they are 3×3 500 m pixels vs 0.5°×0.5° grid aggregated from the 500 m data). We use nearest neighbors to match the ERA-scale grid to the longitude/latitude of individual EC sites. The original site-scale analysis has 68 sites. All are assumed to be C3 plants due to a lack of species information. The ERA-scale analysis distinguishes between C3/C4 plants. In order to compare the site-scale and ERA-scale analyses, we exclude all C4 grids. Also, because some EC sites are located near the coast or water, they are classified as non-vegetation in the coarse-resolution ERA-scale analysis. Therefore, the number of sites compared is reduced to 52.

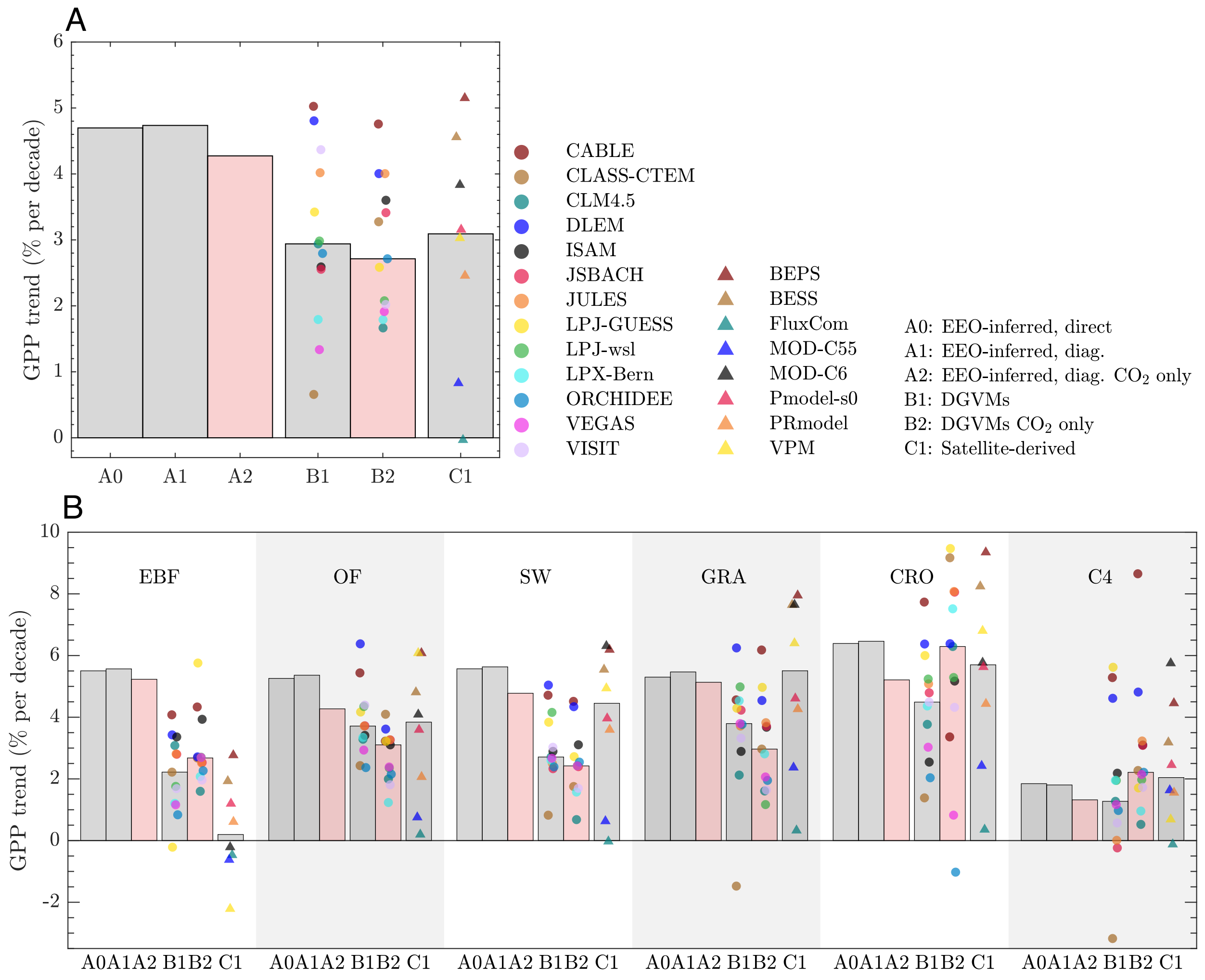


**Fig. S7. Relative GPP trends similar to Fig. 3 but using CRU-JRA55 forcing to estimate the trends in category "A".** Trends are divided by their respective mean GPP from 2001 to 2016. **a,** all biomes. **b,** individual biomes, including evergreen broadleaf forests (EBF), other forests (OF), short woody vegetation (SW), grasslands (GRA), croplands (CRO), and C4 vegetation (C4). "A0": estimated directly from our EEO framework. "A1": diagnostic trends composited from partial derivatives using the EEO framework. "A2": diagnostic $CO_2$-induced trends through the partial derivative approach using the EEO framework. "B1": 13 DGVM models, all forcing time-varying. "B2": 13 DGVM models, $CO_2$ only. "C1": 8 satellite-derived GPP products. For B1, B2, and C1, the bars represent their median. The grey bars represent GPP trends considering all effects. The red bars represent GPP trends caused by $CO_2$ only.

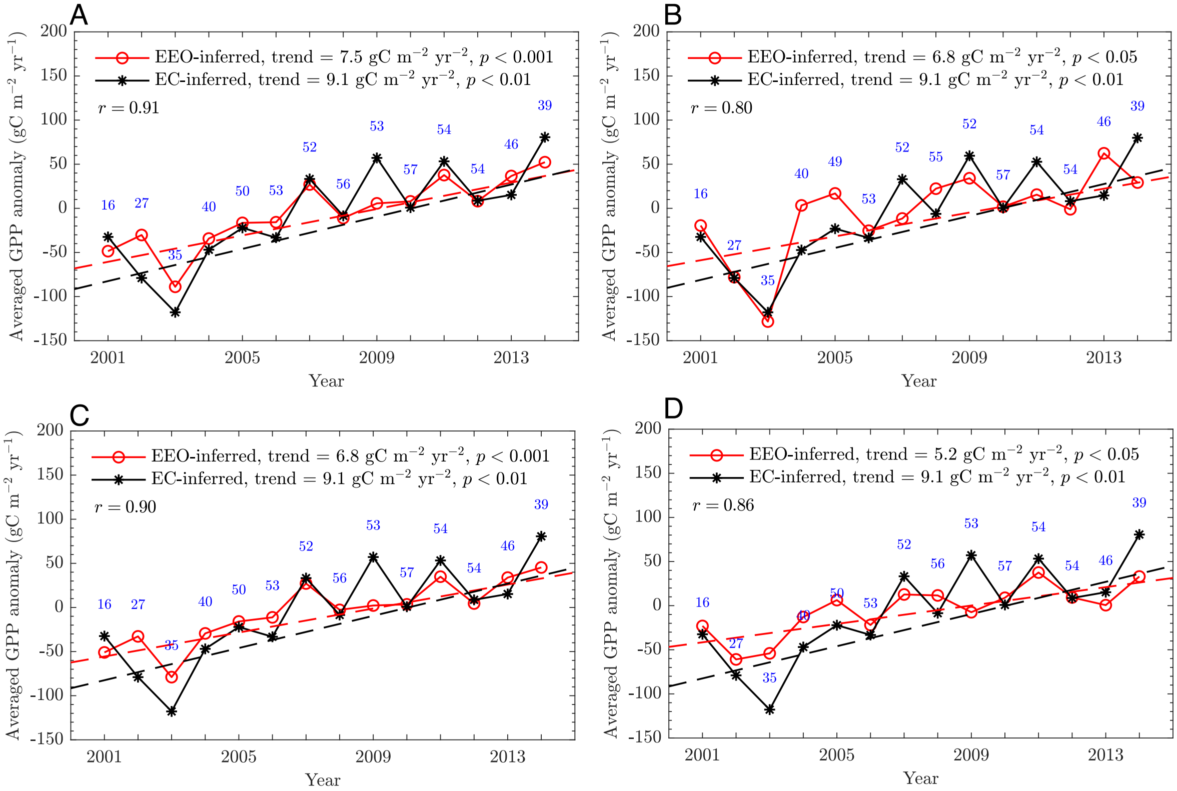


**Fig. S8.** Impact of acclimating photosynthetic capacity at different timescales. All figures are similar to Fig. 1A, but the reference values of photosynthetic capacity adjusted according to (A) year-to-year variation of peak month $CO_2$ + average of peak month meteorological conditions during the study period; (B) year-to-year variation of peak month $CO_2$ and meteorological conditions; (C) month-to-month variation of $CO_2$ + average of peak month meteorological conditions during the study period; and (D) month-to-month variation of $CO_2$ and meteorological conditions.

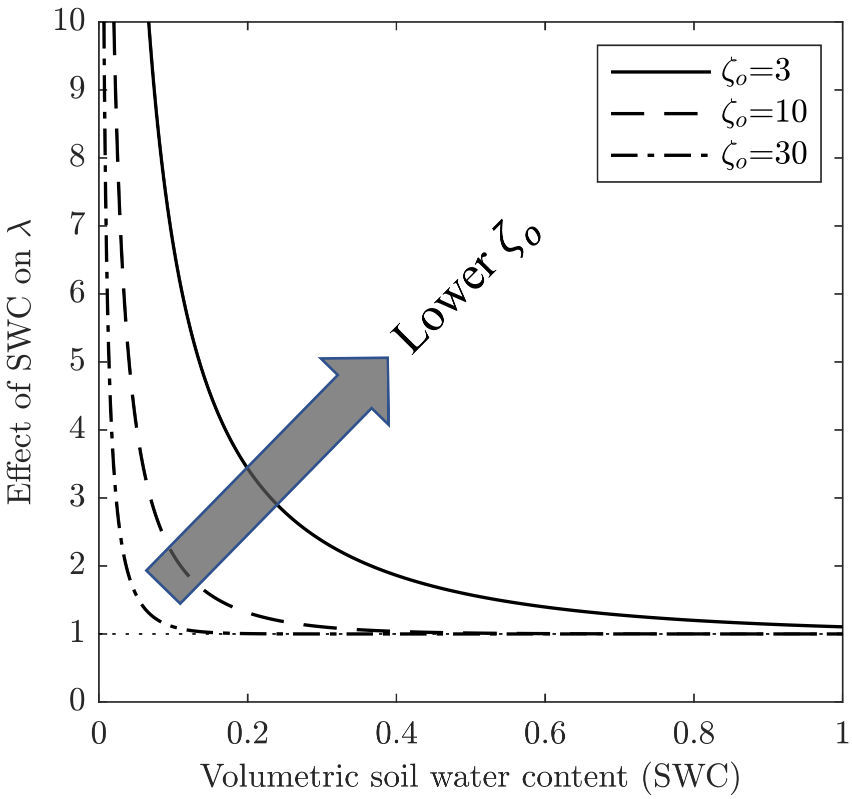


**Fig. S9.** Effect of volumetric soil water content on marginal water use efficiency. A lower $\zeta_o$ indicates a more isohydric plant which tends to vary stomatal conductance with soil moisture. The y-axis represents $F(\zeta_o, SWC)$ in Eq. S33.

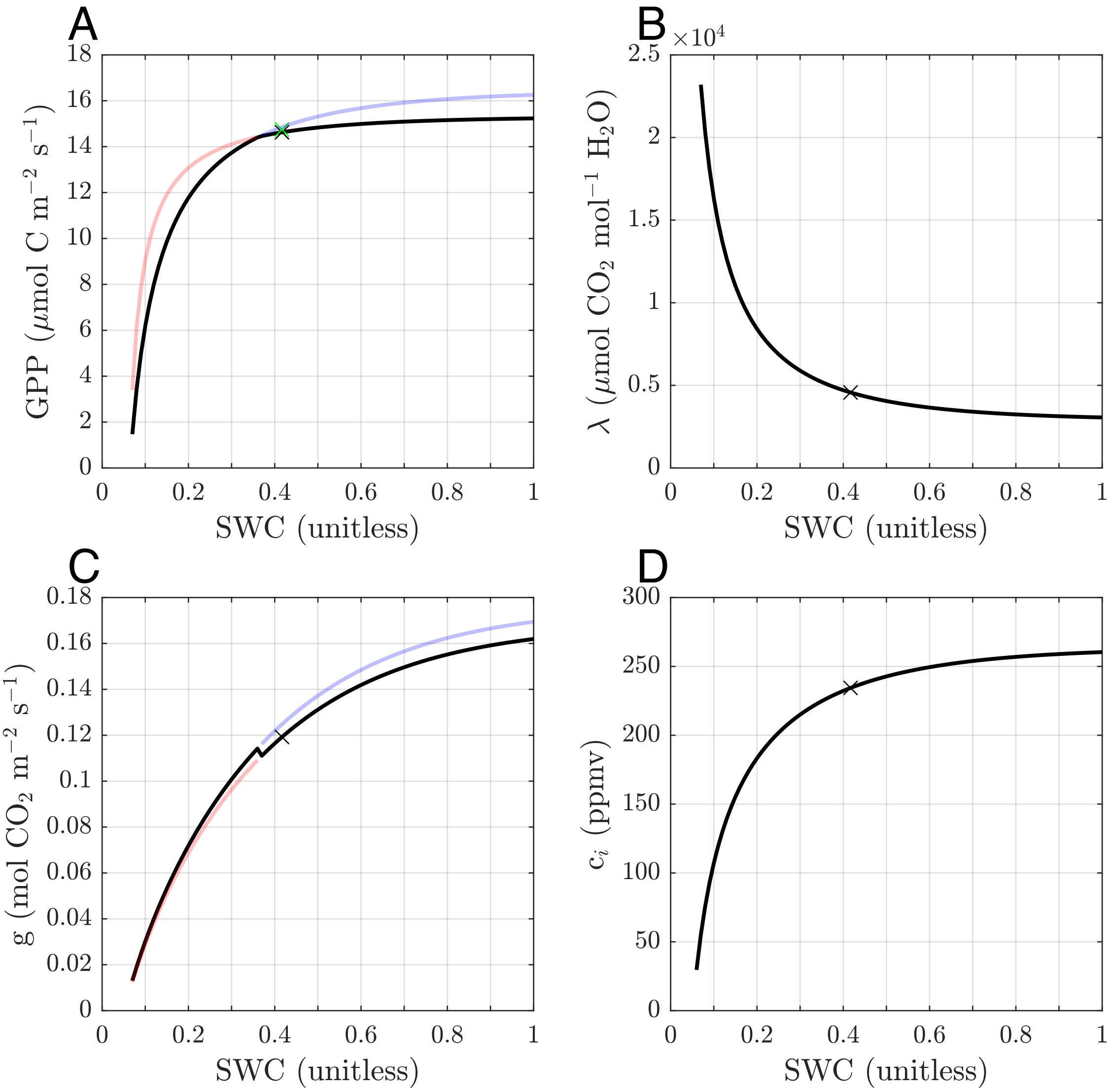


**Fig. S10**. Effects of volumetric soil water content (SWC) on EEO-inferred GPP and other key variables. Black lines show the results of univariate sensitivity response to SWC for (A) canopy-level GPP, (B) marginal water use efficiency, (C) canopy-level stomatal conductance of $CO_2$, and (D) intercellular leaf $CO_2$ concentration. Blue lines indicate extrapolated light-saturated conditions, and red lines indicate extrapolated light-limited conditions. Black '×' represents the simulated values by our framework under the actual conditions, and the green '×' represents the FLUXNET2015 GPP. Example data are selected from the site "US-MMS" measured in May, 2004. This site is classified as a deciduous broadleaf forest. For the measured input variables, $c_a$: 377.36 ppmv, $T_a$: 293.7045 K, LAI: 4.7778 m$^2$ m$^{-2}$, SWC: 0.4169, $q_a$: 0.0107 kg kg$^{-1}$, $P$: 98246.3 Pa, PPFD: 749.1219 μmol m$^{-2}$ s$^{-1}$, with which canopy-scale $V_{cmax}$ is estimated to be 55.9135 μmol m$^{-2}$ s$^{-1}$. The long-term mean $c_i/c_a$ ratio in May is estimated to be 0.665 over 2001-2014. The mean $\left|\frac{\overline{G(\mu)}}{\mu}\right|$ for May is 1.3935. $\zeta_o$ is 3.5807. In the sensitivity analysis, the SWC range is 0 to 1. Low SWC triggers a complete stomatal closure (when SWC<0.07). The discontinuity in **c** at the point of photosynthetic light-limiting transition is due to the neglect of the $CO_2$ compensation point and dark respiration in the derivation of stomatal conductance.

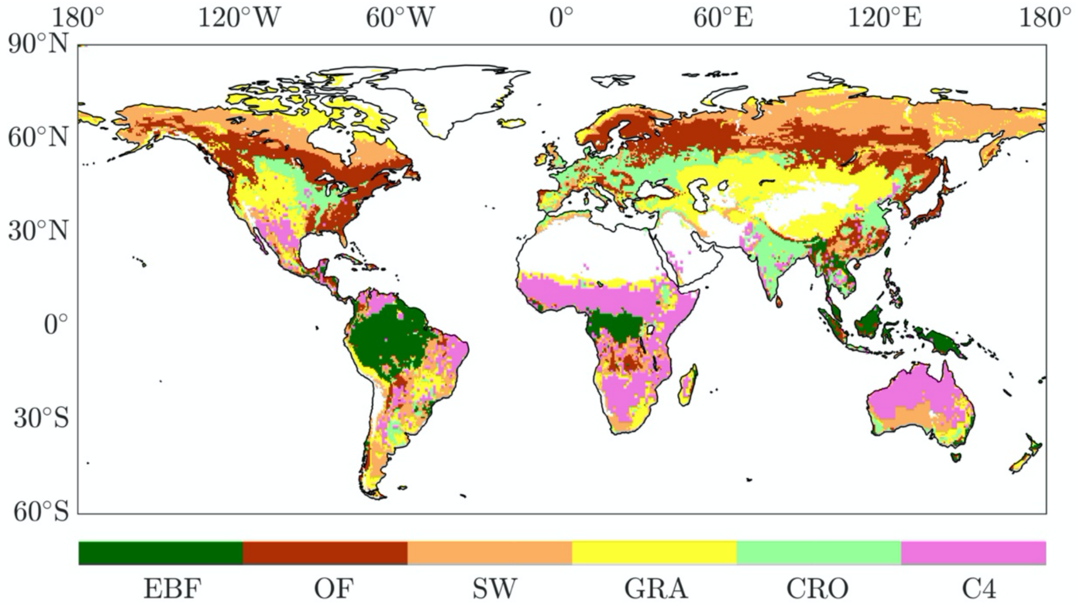


**Fig. S11.** Distribution of biome types according to MODIS. EBF: evergreen broadleaf forests, OF: other forests expect for EBF, SW: Short woody vegetation, GRA: grasslands, CRO: croplands, C4: C4 vegetation.

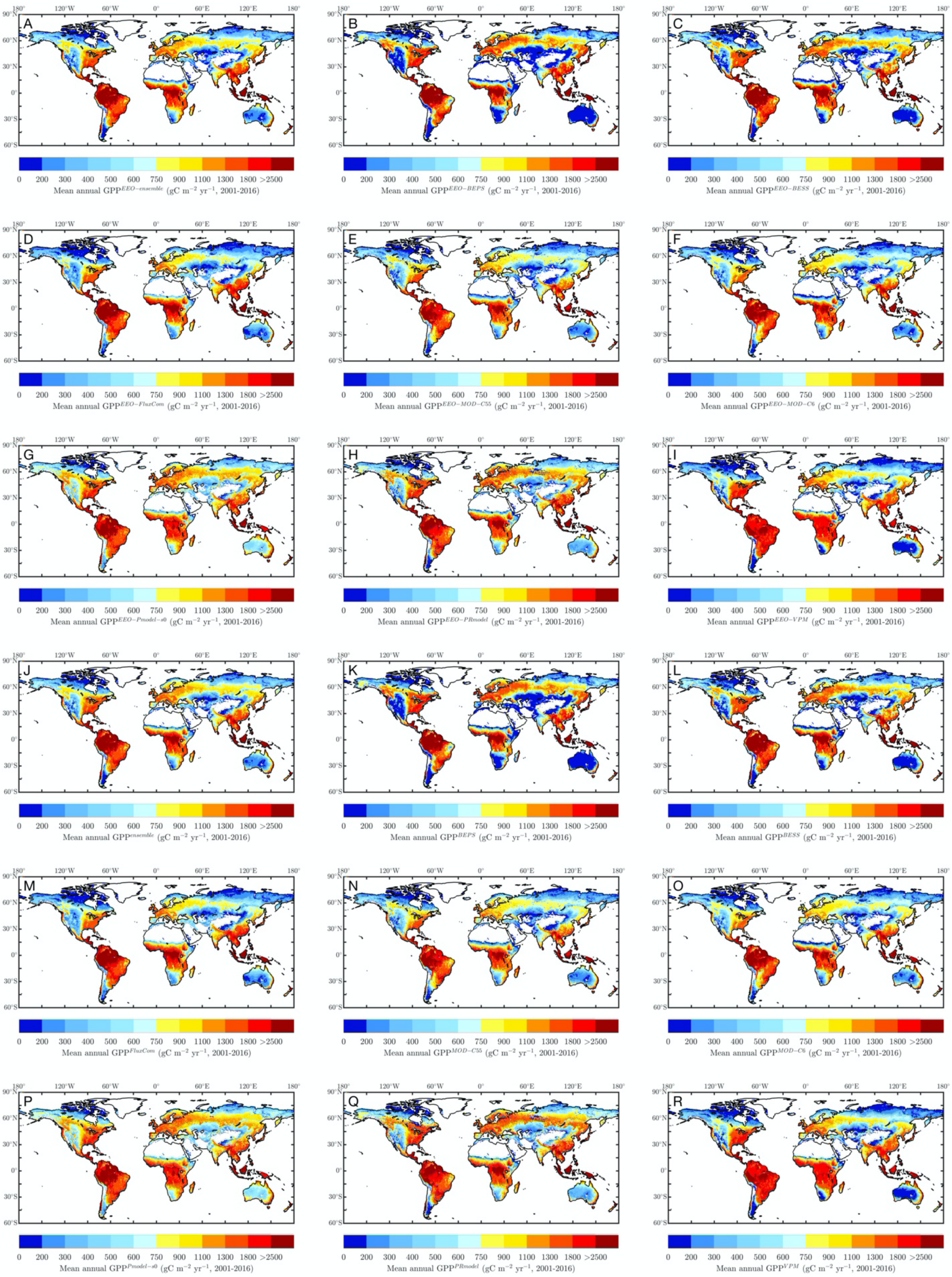
A
Mean annual GPP^EEO-ensemble (gC m^-2 yr^-1, 2001-2016)
B
Mean annual GPP^EEO-BEPS (gC m^-2 yr^-1, 2001-2016)
C
Mean annual GPP^EEO-BESS (gC m^-2 yr^-1, 2001-2016)
D
Mean annual GPP^EEO-FluxCom (gC m^-2 yr^-1, 2001-2016)
E
Mean annual GPP^EEO-MOD-C55 (gC m^-2 yr^-1, 2001-2016)
F
Mean annual GPP^EEO-MOD-C6 (gC m^-2 yr^-1, 2001-2016)
G
Mean annual GPP^EEO-Pmodel-s0 (gC m^-2 yr^-1, 2001-2016)
H
Mean annual GPP^EEO-PRmodel (gC m^-2 yr^-1, 2001-2016)
I
Mean annual GPP^EEO-VPM (gC m^-2 yr^-1, 2001-2016)
J
Mean annual GPP^ensemble (gC m^-2 yr^-1, 2001-2016)
K
Mean annual GPP^BEPS (gC m^-2 yr^-1, 2001-2016)
L
Mean annual GPP^BESS (gC m^-2 yr^-1, 2001-2016)
M
Mean annual GPP^FluxCom (gC m^-2 yr^-1, 2001-2016)
N
Mean annual GPP^MOD-C55 (gC m^-2 yr^-1, 2001-2016)
O
Mean annual GPP^MOD-C6 (gC m^-2 yr^-1, 2001-2016)
P
Mean annual GPP^Pmodel-s0 (gC m^-2 yr^-1, 2001-2016)
Q
Mean annual GPP^PRmodel (gC m^-2 yr^-1, 2001-2016)
R
Mean annual GPP^VPM (gC m^-2 yr^-1, 2001-2016)
0 200 300 400 500 600 750 900 1100 1300 1800 >2500
180° 120°W 60°W 0° 60°E 120°E 180°
90°N 60°N 30°N 0° 30°S 60°S

**Fig. S12.** Spatial distribution of the mean annual GPP during 2001-2016. (A-I) EEO-inferred GPP, calibrated by the ensemble mean of the 8 satellite-derived GPP products, BEPS, BESS, FluxCom, MOD-C55, MOD-C6, Pmodel-s0, PR-model, and VPM, respectively. (J-R) The original satellite-derived GPP products used to calibrate EEO-inferred GPP in A-I.

**Reference**


1. G. Katul, S. Manzoni, S. Palmroth, R. Oren, A stomatal optimization theory to describe the effects of atmospheric $CO_2$ on leaf photosynthesis and transpiration. *Ann Bot-london* 105, 431–442 (2010).

2. S. L. Dingman, *Physical Hydrology* (2008).

3. G. D. Farquhar, S. von Caemmerer, J. A. Berry, A biochemical model of photosynthetic $CO_2$ assimilation in leaves of C3 species. *Planta* 149, 78–90 (1980).

4. S. Manzoni, *et al.*, Optimizing stomatal conductance for maximum carbon gain under water stress: a meta-analysis across plant functional types and climates. *Functional Ecology* 25, 456–467 (2011).

5. C. B. Osmond, *et al.*, "Functional Significance of Different Pathways of $CO_2$ Fixation in Photosynthesis" in Physiological Plant Ecology II: Water Relations and Carbon Assimilation., (1982), pp. 479–547.

6. S. V. Caemmerer, *Biochemical models of leaf photosynthesis* (2000).

7. J. Lloyd, G. D. Farquhar, 13C discrimination during $CO_2$ assimilation by the terrestrial biosphere. *Oecologia* 99, 201–215 (1994).

8. G. G. KATUL, S. Palmroth, R. OREN, Leaf stomatal responses to vapour pressure deficit under current and $CO_2$-enriched atmosphere explained by the economics of gas exchange. *Plant Cell Environ* 32, 968–979 (2009).

9. I. R. Cowan, F. GD, Stomatal function in relation to leaf metabolism and environment. (1977).

10. B. E. Medlyn, R. A. Duursma, M. G. D. Kauwe, I. C. Prentice, The optimal stomatal response to atmospheric $CO_2$ concentration: Alternative solutions, alternative interpretations. *Agr Forest Meteorol* 182, 200–203 (2013).

11. G. G. Katul, R. Oren, S. Manzoni, C. Higgins, M. B. Parlange, Evapotranspiration: A process driving mass transport and energy exchange in the soil-plant-atmosphere-climate system. *Rev Geophys* 50, 1083–25 (2012).

12. S. Manzoni, G. Katul, P. A. Fay, H. W. Polley, A. Porporato, Modeling the vegetation–atmosphere carbon dioxide and water vapor interactions along a controlled $CO_2$ gradient. *Ecol Model* 222, 653–665 (2011).

13. I. C. Prentice, *et al.*, Balancing the costs of carbon gain and water transport: testing a new theoretical framework for plant functional ecology. *Ecology Letters* 17, 82–91 (2013).

14. J.-L. Chen, J. F. Reynolds, P. C. Harley, J. D. Tenhunen, Coordination theory of leaf nitrogen distribution in a canopy. *Oecologia* 93, 63–69 (1993).

15. V. Maire, *et al.*, The Coordination of Leaf Photosynthesis Links C and N Fluxes in C3 Plant Species. *PLOS ONE* 7, e38345-15 (2012).

16. H. Wang, *et al.*, Towards a universal model for carbon dioxide uptake by plants. *Nat Plants* 3, 734–741 (2017).

17. T. F. Keenan, *et al.*, Recent pause in the growth rate of atmospheric $CO_2$ due to enhanced terrestrial carbon uptake. *Nature Communications* 7, 13428–10 (2016).

18. B. D. Stocker, *et al.*, P-model v1.0: an optimality-based light use efficiency model for simulating ecosystem gross primary production. *Geosci Model Dev* 13, 1545–1581 (2020).

19. H. Vogel, The law of the relation between the viscosity of liquids and the temperature. *Phys. Z* 22, 645–646 (1921).

20. K. A. Mott, Do stomata respond to $CO_2$ concentrations other than intercellular? *Plant Physiology* 86, 200 (1988).

21. S. M. Assmann, The cellular basis of guard cell sensing of rising $CO_2$. *Plant, Cell & Environment* 22, 629–637 (1999).

22. E. A. Ainsworth, A. Rogers, The response of photosynthesis and stomatal conductance to rising [$CO_2$]: mechanisms and environmental interactions. *Plant Cell Environ* 30, 258–270 (2007).

23. N. G. Smith, *et al.*, Global photosynthetic capacity is optimized to the environment. *Ecology Letters* 22, 506–517 (2019).

24. N. G. Smith, T. F. Keenan, Mechanisms underlying leaf photosynthetic acclimation to warming and elevated $CO_2$ as inferred from least-cost optimality theory. *Global Change Biology* 26, 5202–5216 (2020).

25. J. Kattge, W. Knorr, Temperature acclimation in a biochemical model of photosynthesis: a reanalysis of data from 36 species. *Plant Cell Environ* 30, 1176–1190 (2007).

26. B. E. Medlyn, *et al.*, Effects of elevated [$CO_2$] on photosynthesis in European forest species: a meta-analysis of model parameters. *Plant, Cell & Environment* 22, 1475–1495 (2002).

27. K. Y. Crous, M. B. Walters, D. S. Ellsworth, Elevated $CO_2$ concentration affects leaf photosynthesis–nitrogen relationships in Pinus taeda over nine years in FACE. *Tree Physiol* 28, 607–614 (2008).

28. X. Luo, T. F. Keenan, Global evidence for the acclimation of ecosystem photosynthesis to light. *Nature Ecology & Evolution* 189, 1–19 (2020).

29. S. von Caemmerer, G. D. Farquhar, Some relationships between the biochemistry of photosynthesis and the gas exchange of leaves. *Planta* 153, 376–387 (1981).

30. , *Physiological Plant Ecology II: Water Relations and Carbon Assimilation* (1982).

31. B. D. Stocker, *et al.*, Drought impacts on terrestrial primary production underestimated by satellite monitoring. *Nat Geosci* 12, 264–270 (2019).

32. Y. Wang, J. S. Sperry, W. R. L. Anderegg, M. D. Venturas, A. T. Trugman, A theoretical and empirical assessment of stomatal optimization modeling. *New Phytologist* 227, 311–325 (2020).

33. Y.-S. Lin, *et al.*, Optimal stomatal behaviour around the world. *Nature Climate Change* 5, 459–464 (2015).

34. V. Humphrey, *et al.*, Sensitivity of atmospheric $CO_2$ growth rate to observed changes in terrestrial water storage. *Nature* 560, 628–631 (2018).

35. V. Humphrey, *et al.*, Soil moisture–atmosphere feedback dominates land carbon uptake variability. *Nature*, 1–18 (2021).

36. D. W. Lawlor, W. Tezara, Causes of decreased photosynthetic rate and metabolic capacity in water-deficient leaf cells: a critical evaluation of mechanisms and integration of processes. *Ann Bot-london* 103, 561–579 (2009).

37. G. E. P. Campos, *et al.*, Ecosystem resilience despite large-scale altered hydroclimatic conditions. *Nature*, 1–5 (2013).

38. C. J. Bernacchi, E. L. Singsaas, C. Pimentel, A. R. P. Jr, S. P. Long, Improved temperature response functions for models of Rubisco-limited photosynthesis. *Plant, Cell & Environment* 24, 253–259 (2001).

39. A. P. Walker, *et al.*, Integrating the evidence for a terrestrial carbon sink caused by increasing atmospheric $CO_2$. *New Phytologist* 229, 2413–2445 (2021).

40. J. M. Chen, *et al.*, Vegetation structural change since 1981 significantly enhanced the terrestrial carbon sink. *Nature Communications*, 1–7 (2019).

41. C. Jiang, Y. Ryu, Multi-scale evaluation of global gross primary productivity and evapotranspiration products derived from Breathing Earth System Simulator (BESS). *Remote Sens Environ* 186, 528–547 (2016).

42. M. Jung, *et al.*, Scaling carbon fluxes from eddy covariance sites to globe: synthesis and evaluation of the FLUXCOM approach. *Biogeosciences* 17, 1343–1365 (2020).

43. S. W. Running, *et al.*, A Continuous Satellite-Derived Measure of Global Terrestrial Primary Production. *BioScience* 54, 547–560 (2004).

44. Y. Zhang, *et al.*, A global moderate resolution dataset of gross primary production of vegetation for 2000–2016. *Sci Data* 4, 170165 (2017).

45. P. Friedlingstein, *et al.*, Global Carbon Budget 2020. *Earth System Science Data* 12, 3269–3340 (2020).
